# *Helicobacter pylori* Treatment and Gastric Cancer Risk Among Individuals With High Genetic Risk for Gastric Cancer

**DOI:** 10.1001/jamanetworkopen.2024.13708

**Published:** 2024-05-29

**Authors:** Heng-Min Xu, Yuting Han, Zong-Chao Liu, Zhou-Yi Yin, Meng-Yuan Wang, Canqing Yu, Jun-Ling Ma, Dianjianyi Sun, Wei-Dong Liu, Yang Zhang, Tong Zhou, Jing-Ying Zhang, Pei Pei, Ling Yang, Iona Y. Millwood, Robin G. Walters, Yiping Chen, Huaidong Du, Zhengming Chen, Wei-Cheng You, Liming Li, Kai-Feng Pan, Jun Lv, Wen-Qing Li

**Affiliations:** 1 State Key Laboratory of Holistic Integrative Management of Gastrointestinal Cancers, Beijing Key Laboratory of Carcinogenesis and Translational Research, Department of Cancer Epidemiology, Peking University Cancer Hospital & Institute, Beijing, China; 2 Department of Epidemiology & Biostatistics, School of Public Health, Peking University, Beijing, China; 3 Peking University Center for Public Health and Epidemic Preparedness & Response, Beijing, China; 4 Key Laboratory of Epidemiology of Major Diseases (Peking University), Ministry of Education, Beijing, China; 5 Key Laboratory of Carcinogenesis and Translational Research (Ministry of Education), Peking University Cancer Hospital & Institute, Beijing, China; 6 Linqu Public Health Bureau, Linqu, Shandong, China; 7 Medical Research Council Population Health Research Unit at the University of Oxford, Oxford, United Kingdom; 8 Clinical Trial Service Unit & Epidemiological Studies Unit (CTSU), Nuffield Department of Population Health, University of Oxford, Oxford, United Kingdom; 9 State Key Laboratory of Vascular Homeostasis and Remodeling, Peking University, Beijing, China

## Abstract

**Importance:**

*Helicobacter pylori* treatment and nutrition supplementation may protect against gastric cancer (GC), but whether the beneficial effects only apply to potential genetic subgroups and whether high genetic risk may be counteracted by these chemoprevention strategies remains unknown.

**Objective:**

To examine genetic variants associated with the progression of gastric lesions and GC risk and to assess the benefits of *H pylori* treatment and nutrition supplementation by levels of genetic risk.

**Design, Setting, and Participants:**

This cohort study used follow-up data of the Shandong Intervention Trial (SIT, 1989-2022) and China Kadoorie Biobank (CKB, 2004-2018) in China. Based on the SIT, a longitudinal genome-wide association study was conducted to identify genetic variants for gastric lesion progression. Significant variants were examined for incident GC in a randomly sampled set of CKB participants (set 1). Polygenic risk scores (PRSs) combining independent variants were assessed for GC risk in the remaining CKB participants (set 2) and in an independent case-control study in Linqu.

**Exposures:**

*H pylori* treatment and nutrition supplementation.

**Main Outcomes and Measures:**

Primary outcomes were the progression of gastric lesions (in SIT only) and the risk of GC. The associations of *H pylori* treatment and nutrition supplementation with GC were evaluated among SIT participants with different levels of genetic risk.

**Results:**

Our analyses included 2816 participants (mean [SD] age, 46.95 [9.12] years; 1429 [50.75%] women) in SIT and 100 228 participants (mean [SD] age, 53.69 [11.00] years; 57 357 [57.23%] women) in CKB, with 147 GC cases in SIT and 825 GC cases in CKB identified during follow-up. A PRS integrating 12 genomic loci associated with gastric lesion progression and incident GC risk was derived, which was associated with GC risk in CKB (highest vs lowest decile of PRS: hazard ratio [HR], 2.54; 95% CI, 1.80-3.57) and further validated in the analysis of 702 case participants and 692 control participants (mean [SD] age, 54.54 [7.66] years; 527 [37.80%] women; odds ratio, 1.83; 95% CI, 1.11-3.05). *H pylori* treatment was associated with reduced GC risk only for individuals with high genetic risk (top 25% of PRS: HR, 0.45; 95% CI, 0.24-0.82) but not for those with low genetic risk (HR, 0.81; 95% CI, 0.50-1.34; *P* for interaction = .03). Such effect modification was not found for vitamin (*P* for interaction = .93) or garlic (*P *for interaction = .41) supplementation.

**Conclusions and Relevance:**

The findings of this cohort study indicate that a high genetic risk of GC may be counteracted by *H pylori* treatment, suggesting primary prevention could be tailored to genetic risk for more effective prevention.

## Key Points

**Question:**

Is genetic susceptibility for gastric cancer associated with the outcomes of its primary prevention strategies?

**Findings:**

In this cohort study of more than 100 000 Chinese adults, a polygenic risk score associated with the risk of gastric cancer was derived. *Helicobacter pylori* treatment was associated with reduced gastric cancer risk for individuals with a high genetic risk during long-term follow-up but not for those with low genetic risk.

**Meaning:**

The findings of this study support the benefits of *H pylori* treatment for individuals with a high genetic risk of gastric cancer and suggest the utilization of genetic susceptibility for implementing effective gastric cancer primary prevention.

## Teaser

This cohort study examines whether outcomes of primary prevention strategies for gastric cancer, including *Helicobacter pylori* treatment, differ by genetic risk profile among adults in China.

## Introduction

Gastric cancer (GC) is a leading cause of cancer-related deaths worldwide.^1^ The process of gastric carcinogenesis involves multistage progression of gastric lesions,^2^ with the risk of developing invasive GC elevating to 17.4-fold for superficial intestinal metaplasia, 29.3-fold for deep intestinal metaplasia or mild dysplasia, and 104.2-fold for moderate-to-severe dysplasia compared with superficial gastritis or chronic atrophic gastritis in our endoscopy-based cohort study.^3^ However, even among individuals with *Helicobacter pylori* infection, gastric lesions display distinct patterns over time, with only some progressing to more advanced stages or GC,^3,4^ indicating possibly multifactorial interplays in the evolution of precancerous gastric lesions and GC development.^5,6^

A heritability of 22% has been previously estimated for GC.^7,8^ Genome-wide association studies (GWASs) of a case-control design have identified multiple genomic loci with predisposition to GC,^9,10,11,12,13,14^ while a prospective study further suggested increased risk of incident GC associated with a high polygenic risk score (PRS).^15^ To our knowledge, few studies have examined genetic predisposition in the context of the multistep cascade progression of gastric carcinogenesis, which may potentially discover genetic variants accounting for gastric lesion progression to GC, yielding new insights into the identification of high-risk populations and optimizing approaches to GC prevention.

Accumulating evidence supports *H pylori* eradication as a means to eliminate GC.^16,17^ The Shandong Intervention Trial (SIT) that we initiated in Linqu County, China, was the first trial of which we are aware to show significantly decreased GC incidence resulting from *H pylori* treatment^6,18,19^ and also demonstrated enduring benefits of nutrition supplementation for GC prevention.^20^ Despite these findings, a one-size-fits-all approach of past strategies may not be biologically optimal considering the complexity of GC etiology. Defining and triaging populations that would derive the most benefit from chemoprevention is crucial to optimizing tailored strategies for effective GC prevention. Given the known genetic predisposition underlying GC, it is important to clarify whether chemoprevention by eradicating *H pylori* or nutrition supplementation is effective for specific genetically susceptible subgroups and whether primary prevention approaches would counteract a high genetic risk for GC.

To fill these knowledge gaps, a longitudinal GWAS was conducted to examine genetic variants associated with the progression of gastric lesions and the risk of incident GC. Applying PRSs to denote GC genetic predisposition, we assessed the associations of *H pylori* eradication and nutrition supplementation with GC risk in individuals with high and low genetic risk. Two population-based prospective studies in China, namely the SIT and China Kadoorie Biobank (CKB), have allowed unique collaborative opportunities to address our goals.

## Methods

The original SIT trial and current analyses as well as the external case-control study were approved by institutional review boards of Peking University Cancer Hospital & Institute. The CKB study was approved by the Ethical Review Committee of the Chinese Center for Disease Control and Prevention and the Oxford Tropical Research Ethics Committee, University of Oxford. All participants provided written informed consent. This report followed the Strengthening the Reporting of Observational Studies in Epidemiology (STROBE) reporting guideline and the Strengthening the Reporting of Genetic Association Studies (STREGA) reporting guideline.

### Study Design

A 2-stage design was used to investigate genetic variants associated with the progression of gastric lesions and risk of incident GC (eFigure 1 in Supplement 1). We initially conducted a longitudinal GWAS for genetic variants associated with the progression of gastric lesions based on the SIT (2816 participants). Genetic variants that displayed promising associations underwent further assessment for the risk of incident GC in a randomly selected group of CKB participants (set 1; 49 912 participants). A PRS combining independent single-nucleotide variants (SNVs) were assessed for GC risk in the remaining CKB participants (set 2; 50 316 participants) and further validated in an external case-control set (1394 participants). Finally, we explored whether *H pylori* treatment and nutrition supplementation were associated with different outcomes among SIT participants with different levels of genetic risk of GC.

### SIT Cohort

Details were described previously.^3,21^ In brief, 4382 residents aged 35 to 64 years were identified in 1989 from 14 villages in Linqu, a rural area in Shandong province, which had one of the highest GC mortalities worldwide.^4^ Of them, 3399 individuals volunteered to undergo gastroscopic examinations and provided peripheral blood for *H pylori* serology. Among them, none had completely normal gastric histopathology. After the exclusion of 13 GCs in this endoscopic screening phase, 3386 participants were prospectively followed up with scheduled endoscopic examinations in 1994 for the evolution of gastric lesions and diagnoses of GC.^3,21^

In 1994, individuals still younger than 65 years, along with other endoscopically screened residents of these villages aged 35 to 64 years, were asked to enroll in a randomized, placebo-controlled trial (NCT00339768).^18,20,22,23^ A total of 3365 participants were included. Of them, 2258 *H pylori*–seropositive participants were randomly assigned to receive 3 interventions on July 23, 1995, including 2-week *H pylori* treatment with amoxicillin and omeprazole and/or 7.3-year garlic supplementation and/or 7.3-year vitamin supplementation or placebo in a 2 × 2 × 2 factorial design. Meanwhile, 1107 *H pylori*–seronegative participants were randomly assigned to receive garlic supplementation and/or vitamin supplementation or placebos in a 2 × 2 factorial design.^18,20,22,23^ Data from pill counts and sampled blood assays showed excellent treatment adherence. For *H pylori* treatment, 90.16% of participants took pills in full accordance with the protocol; only 0.84% had delays of 1 day or more. For vitamin supplementation and garlic supplementation, several surveys reported an average monthly treatment adherence of 86% to 94% for participants taking all pills. After initial treatment, 382 individuals had treatment failure and received another 2-week retreatment.

Participants were continuously followed up until August 31, 2022. Each participant had gastric histopathologic diagnoses in 1989, 1994, 1999, 2003, and 2022. For this study, 2924 participants had DNA samples meeting the requirement of genotyping platform, and 2816 had genotype data passing quality control (eMethods and eFigure 2 in Supplement 1), with 2604 attending the intervention trial, including 1853 *H pylori*–seropositive and 751 seronegative individuals (eTable 1 in Supplement 1).

### CKB Cohort

Details were described previously.^24,25,26^ Briefly, during baseline survey (June 25, 2004, to July 15, 2008), a total of 512 723 adults aged 30 to 79 years were enrolled from 10 regions in China, including 5 urban and 5 rural areas. A total of 105 408 individuals were initially genotyped,^26^ with 100 639 retained after quality control (eMethods and eFigure 2 in Supplement 1). We additionally excluded self-reported cancers at baseline, leaving 100 228 eligible participants, who were further randomly divided into set 1 (49 912 participants) and set 2 (50 316 participants). Participants lost to follow-up accounted for less than 1% until December 31, 2018.

### Case-Control Validation Set

Genetic data of a case-control study, including 702 GC case participants and 692 control participants aged 30 to 75 years from Linqu county (eMethods in Supplement 1), were used for external validation of PRS associated with GC risk. This ongoing case-control study was designed to examine multiomic signatures for GC, with genotyping of peripheral blood leukocyte DNA samples completed recently. Invasive GC cases were diagnosed between January 2011 and December 2022. Control participants were selected in 2023 from Linqu residents attending physical examination and upper gastrointestinal cancer screening. Participants did not receive interventions for *H pylori* eradication or nutrition supplementation previously.

### Statistical Analysis

Details are shown in the eMethods and eFigure 3 in Supplement 1. Based on repeated gastric histopathological diagnoses of the SIT, we defined the progression of gastric lesions as having increased gastric histopathologic score over time while also considering the rate of change. This was achieved through longitudinal GWAS using a generalized linear mixed model (GLMM; MAGEE package in R),^27,28^ which is known for its conservative approach by considering within-participant correlations.^29^ The effect estimate of the SNV × time interaction term illustrates the association of SNVs with the progression of gastric lesions over time.^28^ SNVs with *P* < .0005 for gastric lesion progression were examined for the risk of incident GC, defined as newly occurring invasive GC cases during follow-up, based on set 1 of the CKB using the SAIGE package in R.^30^ A clumping procedure was used to identify independent signals of genomic loci, and a lead (index) SNV (having the lowest GC association *P* value and linkage disequilibrium *r*^2^ > 0.2 with other SNVs) was defined for each locus. Combining all lead SNVs, a PRS was generated by weighting the dosage of the effect allele for each SNV. The Fine-Gray models accounting for death from causes other than GC as competing risk were used to calculate the hazard ratios (HRs) and 95% CIs for the associations between PRS and GC risk in the CKB and SIT cohorts. Logistic regression models were used to calculate the odds ratios (ORs) and 95% CIs for the PRS associated with GC in the case-control set.

To evaluate the performance of the PRS in discriminating GC risk, the concordance index (*C* index) for the continuous PRS was calculated,^31^ with its robustness evaluated by the gradient boosting method combined with bootstrapping strategy. The appropriate PRS cutoff for classifying high and low genetic risk was determined by assessing PRS percentiles in 5–percentage point increments from 50% to 95%, with the percentile with the highest *C* index adopted as the benchmark threshold.

The χ^2^ test was used to compare *H pylori* eradication rate between high and low genetic risk groups. The Fine-Gray models were used to examine the associations of *H pylori* treatment and nutrition supplementation with GC by different genetic risks in the SIT cohort. For each analysis, *P* values for interaction between the PRS and assessed intervention were calculated. For each intervention, absolute risk reduction (ARR) in GC incidence and the number of participants needed to treat (NNT) to prevent 1 GC over 27.1 years’ follow-up were calculated.^32,33^

For the GLMM, sensitivity analyses were conducted by adjusting for baseline histopathological diagnosis (1989) as a covariate, while utilizing the other 4 diagnosis times (1994, 1999, 2003, and 2022) in the longitudinal matrix or by additionally adjusting for 3 chemoprevention strategies. For the association of PRS with GC, a sensitivity analysis without adjusting for principal components (PCs) was conducted. For the association of interventions by genetic risk, we also performed sensitivity analyses by defining individuals with PRS greater than the threshold incrementally by 5 percentage points from 50% to 95% as having high genetic risk or by excluding 3 SNVs that had minor allele frequency or 2% of less in the SIT or without adjusting for PCs.

A 2-tailed *P* < .05 was considered statistically significant for statistical analyses other than the longitudinal GWAS. Analyses were performed using R version 4.2.2 (R Project for Statistical Computing) or PLINK version 1.9.

## Results

The mean (SD) age at participant enrollment was 46.95 (9.12) years for the 2816 participants in SIT, 53.69 (11.00) years for the 100 228 participants in CKB, and 54.54 (7.66) years for the 1394 participants in the case-control validation set. Women accounted for 50.75% (1429 participants), 57.23% (57 357 participants), and 37.80% (527 participants) in these datasets, respectively (eTable 2 in Supplement 1).

### Longitudinal GWAS in SIT and Examination for GC Risk in CKB

**Figure 1.**
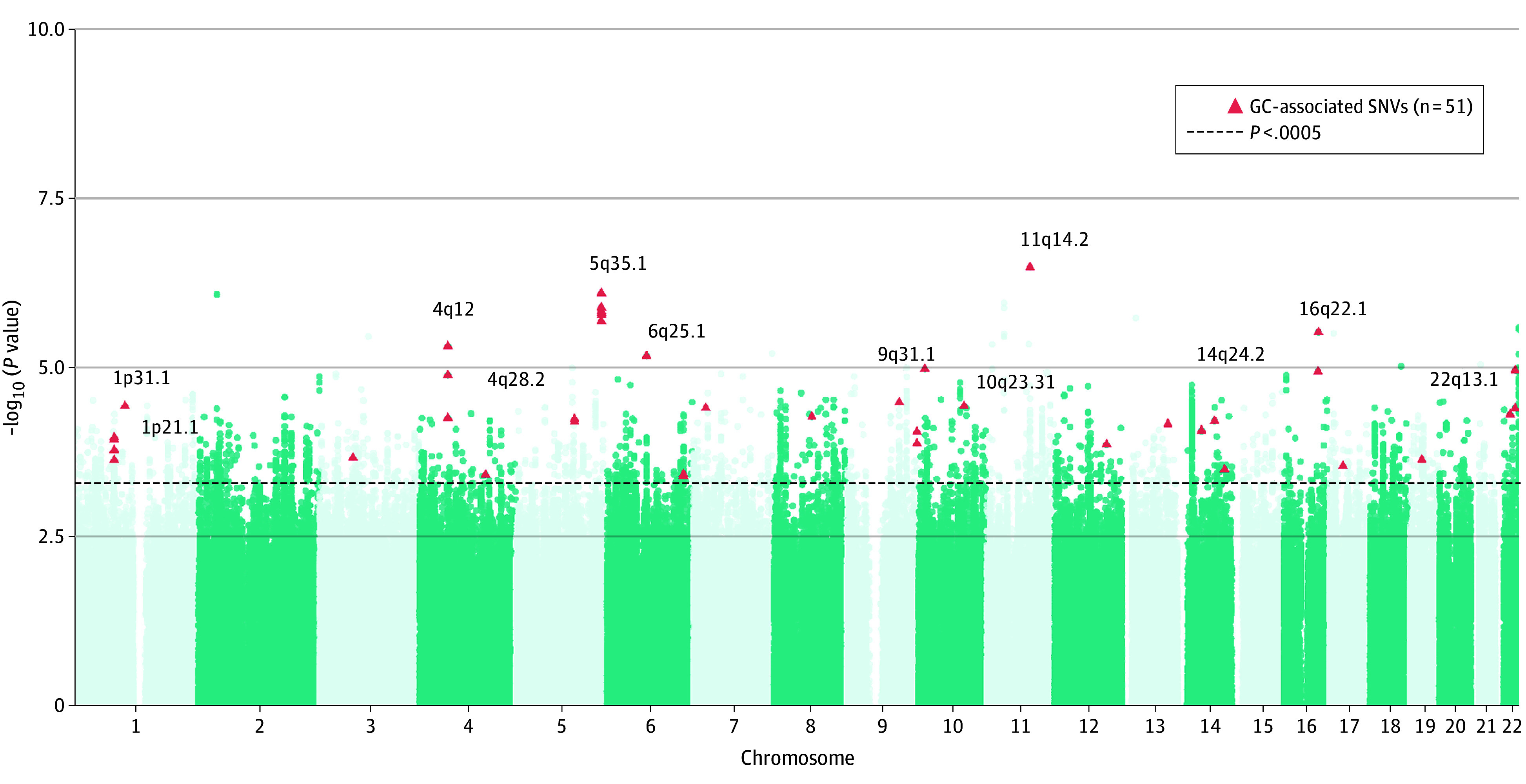
Manhattan Plot for Longitudinal Genome-Wide Association Analysis Each point on the graph represents a variant. The dashed horizontal line indicates the suggestive significance threshold (*P* = .0005) in the Shandong Intervention Trial cohort. The triangles highlight 51 single-nucleotide variants (SNVs) in set 1 of the China Kadoorie Biobank that were further associated with gastric cancer risk (*P* < .05). Overall, 12 genomic loci within gastric cancer–associated SNVs are noted, referred by Annotate Variation cytoband database.

Individuals had a maximum of 5 gastric histopathological diagnoses in the SIT (eTable 3 and eFigure 4 in Supplement 1). The longitudinal GWAS indicated little evidence on confounding of population structure (λ = 1.037) (eFigure 5 in Supplement 1). A total of 4796 SNVs were associated with the progression of gastric lesions at *P* < .0005, but none met the genome-wide Bonferroni-corrected significance threshold (*P* < 5 × 10^−8^) (Figure 1). Of them, 51 SNVs were nominally associated with incident GC risk at *P* < .05 in set 1 of the CKB. A clumping procedure identified 12 independent signals of genomic loci (eTable 4 in Supplement 1). Among prior GWAS catalog–reported significant loci for GC, 5 SNVs (rs6897169 [*PRKAA1*]; rs16893741 and rs2267637 [*PPP1R10*]; rs9461366 [*ZNF204P*/*VN1R10P*]; and rs999197) were associated with the progression of gastric lesions in SIT, and 16 SNVs were associated with GC risk in set 1 of the CKB at *P* < .05 (eTable 5 in Supplement 1). Sensitivity analyses that adjusted for baseline gastric lesions or additionally adjusted for 3 chemoprevention strategies yielded no material change in findings (eTable 6 in Supplement 1). We integrated 12 lead SNVs on key genomic loci to derive a PRS for GC: (0.36 × *rs*12070840) − (0.47 × *rs*78078728) + (0.39 × *rs*41481345) − (0.34 × *rs*2391536) − (0.17 × *rs*6879467) + (0.77 × *rs*9478852) + (0.45 × *rs*139371995) − (0.37 × *rs*7910150) + (0.18 × *rs*2125363) − (0.67 × *rs*10147214) − (1.10 × *rs*1110549) − (0.19 × *rs*5995654).

We also considered the PRS by Jin et al^15^ that integrated 112 SNVs. Due to distinct panels of genotyping arrays, only 82 SNVs were genotyped and imputed in the SIT cohort; we therefore were not able to examine their PRS in its entirety further.

### PRS and GC Risk

During the follow-up period, we recorded a total of 147 incident GC cases in SIT, 375 cases in CKB set 1 and 450 cases in CKB set 2. There was a trend toward an increased risk of developing GC across PRS deciles in the SIT cohort (HR, 1.69; 95% CI, 1.47-1.94; *P* < .001), CKB set 1 (HR, 1.47; 95% CI, 1.35-1.60; *P* < .001) and set 2 (HR, 1.13; 95% CI, 1.03-1.24; *P* = .009) (eFigure 6 in Supplement 1). From the lowest to highest decile of PRS, the cohort age-adjusted incidence rates of GC increased from 39 to 98 per 100 000 person-years in the combined CKB cohort, with an HR of 2.54 (95% CI, 1.80-3.57) for the highest decile (Table 1). The approximately linear, upward-sloping restricted cubic spline curve also demonstrated a positive association between PRS and GC risk (eFigure 7 in Supplement 1). A significant association between PRS and GC risk was also observed in the case-control validation set, with the odds of GC increased to 1.83-fold (95% CI, 1.11-3.05) for the highest PRS decile compared with the lowest decile (Table 1). Sensitivity analysis by removing PCs from covariates showed no material change in the associations (eTable 7 in Supplement 1). To evaluate the accuracy of PRS in estimating GC risk, the gradient boosting combined with bootstrapping strategy achieved a *C* index at 0.79 (95% CI, 0.77-0.82) in CKB set 1, 0.71 (95% CI, 0.69-0.73) in CKB set 2, 0.76 (95% CI, 0.74-0.78) in total CKB, 0.79 (95% CI, 0.74-0.83) in SIT, and 0.82 (95% CI, 0.80-0.84) in the case-control set.

**Table 1.** Deciles of Polygenic Risk Scores and Risk of Gastric Cancer

Decile	SIT				CKB										External case-control validation set		
	No. of cases (PYs)	Age-adjusted IR/100 000 PYs	HR (95% CI)^a^	*P* value	Set 1			Set 2			Total				No. of cases/controls	OR (95% CI)^b^	*P* value
					No. of cases (PYs)	HR (95% CI)^a^	*P* value	No. of cases (PYs)	HR (95% CI)^a^	*P* value	No. of cases (PYs)	Age-adjusted IR/100 000 PYs	HR (95% CI)^a^	*P* value			
1	7 (8758)	89	1 [Reference]	NA	12 (61 222)	1 [Reference]	NA	35 (60 293)	1 [Reference]	NA	47 (121 515)	39	1 [Reference]	NA	63/77	1 [Reference]	NA
2	7 (7720)	82	1.13 (0.40-3.22)	.82	25 (55 139)	2.24 (1.13-4.46)	.02	47 (55 597)	1.43 (0.92-2.21)	.11	71 (110 417)	65	1.63 (1.13-2.36)	.009	75/74	1.43 (0.87-2.35)	.16
3	12 (7785)	159	1.83 (0.72-4.63)	.20	26 (66 064)	1.93 (0.97-3.83)	.06	56 (66 820)	1.43 (0.94-2.18)	.10	83 (133 203)	62	1.56 (1.09-2.23)	.02	80/85	1.19 (0.73-1.93)	.48
4	10 (8280)	134	1.53 (0.58-4.03)	.39	33 (48 322)	3.29 (1.70-6.44)	<.001	32 (49 529)	1.12 (0.70-1.81)	.63	65 (97 151)	67	1.67 (1.15-2.47)	.007	49/55	1.35 (0.78-2.33)	.29
5	7 (7667)	104	1.15 (0.40-3.30)	.79	41 (60 178)	3.38 (1.78-6.44)	<.001	44 (61 320)	1.23 (0.79-1.92)	.36	85 (122 199)	70	1.78 (1.25-2.54)	.002	75/64	1.54 (0.93-2.56)	.10
6	15 (7883)	242	2.30 (0.93-5.69)	.07	30 (55 046)	2.71 (1.38-5.33)	.004	41 (54 194)	1.37 (0.88-2.14)	.17	72 (109 729)	65	1.71 (1.19-2.47)	.004	71/76	1.38 (0.84-2.28)	.21
7	12 (8044)	186	1.79 (0.70-4.56)	.22	43 (57 670)	3.76 (1.98-7.15)	<.001	44 (57 874)	1.25 (0.80-1.97)	.32	84 (114 689)	73	1.89 (1.32-2.70)	<.001	69/62	1.48 (0.89-2.48)	.13
8	15 (8081)	165	2.43 (1.00-5.98)	.05	48 (60 748)	4.07 (2.17-7.64)	<.001	51 (65 090)	1.41 (0.92-2.17)	.12	101 (126 203)	80	2.08 (1.47-2.95)	<.001	64/76	1.16 (0.70-1.92)	.57
9	27 (7778)	372	4.07 (1.77-9.39)	<.001	56 (54 105)	5.20 (2.78-9.71)	<.001	49 (51 438)	1.71 (1.11-2.64)	.02	105 (105 543)	98	2.59 (1.84-3.66)	<.001	75/64	1.88 (1.13-3.13)	.02
10	35 (7612)	486	5.61 (2.49-12.67)	<.001	61 (56 963)	5.38 (2.89-10.00)	<.001	51 (57 578)	1.57 (1.02-2.41)	.04	112 (114 541)	98	2.54 (1.80-3.57)	<.001	81/59	1.83 (1.11-3.05)	.02
Per 1 SD score	147 (79 608)	199	1.69 (1.47-1.94)	<.001	375 (575 456)	1.47 (1.35-1.60)	<.001	450 (579 734)	1.13 (1.03-1.24)	.009	825 (1 155 190)	71	1.28 (1.20-1.36)	<.001	702/692	1.14 (1.02-1.28)	.02

Abbreviations: CKB, China Kadoorie Biobank; HR, hazard ratio; IR, incidence rate; NA, not applicable; OR, odds ratio; PY, person-year; SIT, Shandong Intervention Trial.

^a^ Analyses were conducted using Fine-Gray models accounting for death from causes other than gastric cancer as competing risk, adjusting for age, sex, *Helicobacter pylori* infection status (for SIT only), regions (for CKB only), and principal components.

^b^ Analysis was conducted using logistic regression model, adjusting for age, sex, *H pylori* infection status, and principal components.

### Chemoprevention and GC by Genetic Risk

**Figure 2.**
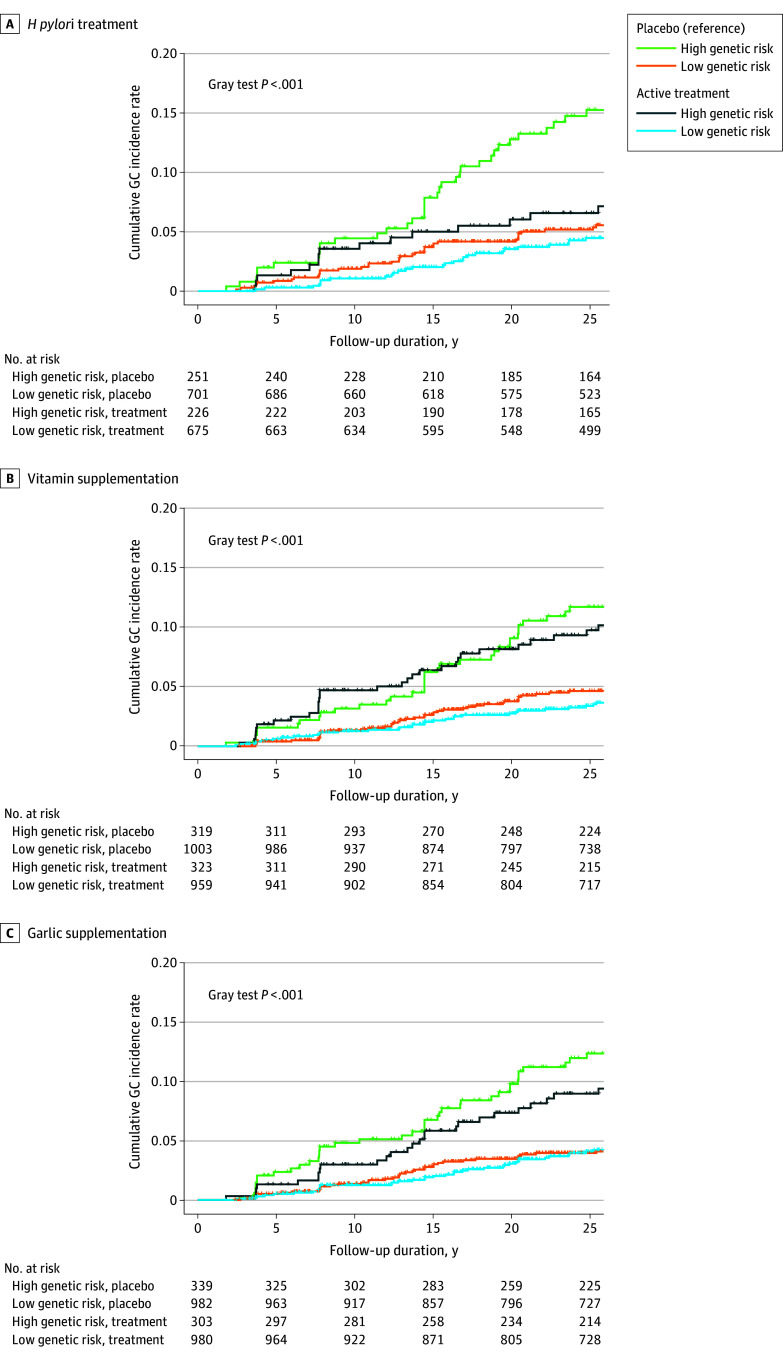
Cumulative Risk of Incident Gastric Cancer Associated With *Helicobacter pylori* Treatment and Nutrition Supplementation by Different Levels of Genetic Risk The Gray test was performed to compare the overall difference in cumulative gastric cancer incidence between individuals with high or low genetic risk receiving active treatment or placebo in each intervention arm.

During the 27.1-year follow-up period after trial randomization of SIT, we documented 139 GC cases (1995-2022). The 75th percentile of PRS was set as the cutoff for classifying genetic risk given its optimal *C* index in comparison with other cutoffs (eFigure 8 in Supplement 1). We therefore defined individuals with the top quartile of PRS as having a high genetic risk for GC. Similar eradication rates of *H pylori* were found between high and low genetic risk groups (358 of 477 [75.05%] vs 1087 of 1376 [79.00%], *P* = .22). In examining active treatment (vs placebo) within the intention-to-treat analyses, *H pylori* treatment was associated with a decreased GC risk only among individuals with a high genetic risk (Figure 2 and Table 2). Compared with participants receiving placebo, *H pylori* active treatment was associated with reduced risk of GC for participants carrying a high genetic risk (HR, 0.45; 95% CI, 0.24-0.82; ARR, 7.75%; NNT, 12.90) but not for those with a low genetic-risk (HR, 0.81; 95% CI, 0.50-1.34; ARR, 1.05%; NNT, 95.24) (*P *for interaction = .03). Significant interactions by PRS were not found for vitamin (*P *for interaction = .93) or garlic (*P *for interaction = .41) supplementation among overall trial participants (Figure 2 and Table 2). Stratified analysis by baseline *H pylori* infection did not reveal a differential association of these interventions by genetic risk (eFigure 9 in Supplement 1). We tested the proportional subdistribution hazards assumption and found no violations (eFigure 10 in Supplement 1).

**Table 2.** Association of *Helicobacter pylori* Treatment and Nutrition Supplementation With Risk of Incident Gastric Cancer by Genetic Risk, Based on the Shandong Intervention Trial

Genetic risk level	No. of cases (person-years)		HR (95% CI)^a^	ARR, %^b^	NNT^b^	*P* for interaction^c^
	Placebo	Treatment				
***H pylori* treatment**						
High	35 (5738)	15 (5300)	0.45 (0.24-0.82)	7.75	12.90	.03
Low	36 (16 846)	28 (16 206)	0.81 (0.50-1.34)	1.05	95.24	
**Vitamin supplementation**						
High	34 (7461)	30 (7393)	0.85 (0.51-1.42)	1.21	82.72	.93
Low	42 (23 862)	33 (23 245)	0.84 (0.53-1.33)	0.89	111.99	
**Garlic supplementation**						
High	38 (7746)	26 (7107)	0.72 (0.43-1.22)	2.98	33.50	.41
Low	38 (23 491)	37 (23 617)	1.02 (0.65-1.62)	0.14	723.15	

Abbreviations: ARR, absolute risk reduction; HR, hazard ratio; NNT, number needed to treat.

^a^ Analyses were conducted using Fine-Gray models accounting for death from causes other than gastric cancer as competing risk, adjusting for age, sex, *H pylori* infection status, and principal components.

^b^ ARR and NNT were calculated for the entire follow-up period (27.1 years) of the Shandong Intervention Trial.

^c^ *P* values for interaction were obtained by incorporating a multiplicative term of the examined intervention and genetic risk variables into the Fine-Gray models.

We further tested the association of combined interventions of *H pylori* treatment and vitamin supplementation for *H pylori*–seropositive participants (eFigure 11 in Supplement 1). Compared with individuals only receiving placebos, a lowered GC incidence was found for individuals with high genetic risk receiving *H pylori* treatment alone, but not for those receiving vitamin supplementation or for those with low genetic risk receiving any active treatment. Sensitivity analyses using different PRS thresholds for high genetic risk (eFigure 8 in Supplement 1), excluding SNVs with a minor allele frequency of 2% or less, or removing PCs from covariates did not materially change the findings (eTable 8 in Supplement 1).

### Functional Annotation

Our in-house RNA-seq data analyses revealed 123 genes expressed in gastric tissues (eTable 9 and eFigure 12 in Supplement 1) that had strong enrichment in the Rap1, Wnt, MAPK, and Ras pathways (eTable 10 in Supplement 1). Overall, 8 genes were identified as differentially expressed genes (DEGs) between GC tumor and nontumor tissues (eTable 9 in Supplement 1), with upregulated *CDH3* and *BAIAP2L2* and downregulated *TMED6* also observed in advanced vs mild gastric lesions (eTable 11 in Supplement 1).

**Figure 3.**
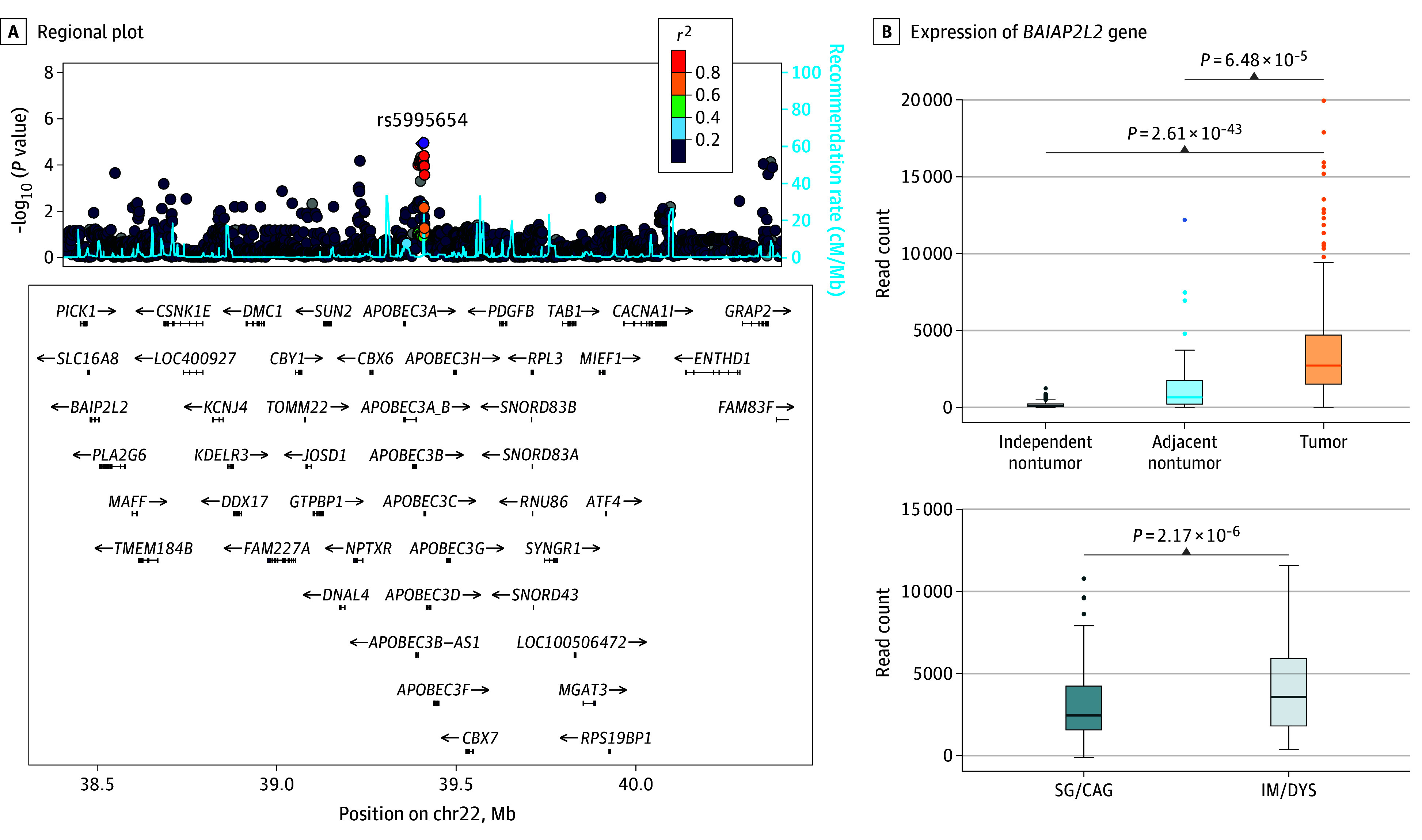
Functional Annotation of 22q13.1 A, A regional plot was used to display positional annotation of gastric cancer–associated variants and *BAIAP2L2* genes. Each point on the graph represents a variant. Colored points represent single-nucleotide variants in different linkage disequilibrium correlation with the lead variant, rs5995654, of 22q13.1. B, Expression of the *BAIAP2L2* gene in different stomach histopathological groups. The boxplot shows a higher expression of *BAIAP2L2* gene in gastric cancer tissue (The Cancer Genome Atlas-Stomach Adenocarcinoma [TCGA-STAD]; n = 413), compared with adjacent nontumor tissue (TCGA-STAD; n = 36) or independent nontumor tissue (Genotype-Tissue Expression; n = 174). The expression of the *BAIAP2L2* gene was higher in advanced gastric lesions (n = 34) than mild gastric lesions (n = 54) in the analysis only among individuals in Linqu without tumors (n = 88). Each box represents the IQR, with the bottom and top edges of the boxes indicating the 25th and 75th percentiles, respectively. The center line denotes the median value. Whiskers show the range, typically 1.5 times the IQR from the quartile edges, highlighting outliers as dots beyond the whiskers. CAG indicates chronic atrophic gastritis; DYS, dysplasia; IM, intestinal metaplasia; SG, superficial gastritis.

Among highlighted DEGs, the cis-expression of *BAIAP2L2* (22q13.1) in nontumor stomach tissues was associated with several expression quantitative trait loci (eQTLs) (eTable 12 in Supplement 1). For the genomic locus of 22q13.1 (chr22:39369272-39408841) (Figure 3A), rs5995654 was designated as the lead (index) SNV. However, combining regulations from RegulomeDB and HaploReg and indicators representing eQTLs to determine potential functional variants, we only derived a prioritization score of 3 for rs5995654, indicating less functional significance, while rs9607601 had the highest prioritization (a score of 10) as a regulatory element (RegulomeDB score of 1b) (eFigure 13 and eTable 13 in Supplement 1). *BAIAP2L2* was overexpressed in GC vs adjacent nontumor tissues (*P* = 6.48 × 10^−5^) or independent nontumor tissues (*P* = 2.61 × 10^−43^) and in advanced vs mild gastric lesions (*P* = 2.17 × 10^−6^) (Figure 3B). Carriers of rs9607601 T-allele were associated with decreased *BAIAP2L2* expression in stomach tissues and blood and reduced risk for gastric lesion progression (SIT: *P* = 5.11 × 10^−4^) and incident GC (CKB: *P* = .009) compared with C-allele (eTable 13 in Supplement 1). Based on RoadMap, rs9607601 is located at the center of DNase I hypersensitivity site peaks and within regions harboring promoter or enhancer histone marks in stomach tissues (eFigure 14 in Supplement 1). Therefore, the rs9607601 T-allele may be associated with decreased GC risk by downregulating *BAIAP2L2* expression. Interestingly, the inverse association of *H pylori* treatment with GC risk was prominent only for the rs9607601 CC genotype (HR, 0.35; 95% CI, 0.18-0.67) but not for CT/TT genotype (HR, 0.94; 95% CI, 0.56-1.56; *P *for interaction = .002) (eTable 14 in Supplement 1).

## Discussion

Based on population-based prospective studies in China, our study corroborated the genetic predisposition underlying cascade evolution of gastric carcinogenesis. The inverse association of *H pylori* treatment with GC risk was observed among individuals with PRSs in the top 25%.

*H pylori* treatment has been recommended by multiple consensus reports and guidelines for GC prevention.^34,35,36,37,38^ Instead of implementing a one-size-fits-all primary prevention approach, limited evidence is yet available for the optimization of targeted populations that may benefit the most. In addition to treatment adherence and antibiotic resistance,^39^ treatment effectiveness may be associated with numerous factors. For treatment-adherent individuals with confirmed *H pylori* antibiotic susceptibility, successful or failed eradication may be affected by lifestyle^40^ and genetic^41^ characteristics. Metabolizer phenotypes of *CYP2C19* were significantly associated with *H pylori* eradication failure.^41^ Even for individuals with successful eradication, long-term benefits on GC risk were only observed for a proportion of individuals,^20^ suggesting the possibly contributory role of genetic and environmental factors. In our study, although the identified genetic variants were not able to distinguish successful eradication from failed treatment, the findings regarding the interactions of genetic predisposition of GC with the long-term outcomes of *H pylori* eradication in intention-to-treat analyses highlight the complex interplays of genetic and environmental factors in GC carcinogenesis.

Functional annotation implied potential biological importance of *BAIAP2L2* (22q13.1) for GC. *BAIAP2L2* is an epithelial-specific BAR domain protein closely related to cell migration,^42^ the overexpression of which may promote GC carcinogenesis via regulating AKT/mTOR and Wnt3a/β-catenin pathways.^43^ Our study confirmed *BAIAP2L2* overexpression in advanced gastric lesions and further upregulation in GC tissues. Its cis-eQTL, rs9607601, as a potential causal variant, was associated with the reduced risk of GC after of *H pylori* treatment, stressing that susceptibility in biologically plausible genes may be integrated for GC risk assessment and risk-tailored prevention.

Recent literature has underscored the role of genetic predisposition in the context of *H pylori* infection, such as pathogenic variants in homologous-recombination genes, concerning GC risk.^8,44^ We conducted quantitative risk assessment of GC based on longitudinal study and long-term cohort analysis and connected the genetic underpinnings of GC with the effectiveness of *H pylori* treatment. Our study supports the potential effect of *H pylori* treatment when genetic predisposition is present, also indicating that selected loci may interact with host molecules involved in response to *H pylori* infection or its consequences. For example, several enriched pathways with the highlighted loci, including Wnt/β-catenin, Ras, EGFR, and MAPK signaling, may get activated by *H pylori* virulence factor CagA,^45^ essential for gastric carcinogenesis. Further mechanistic studies are warranted to elucidate the biological mechanisms of highlighted genetic variants underlying *H pylori–*related GC development.

### Limitations

We acknowledge several limitations. First, heterogeneity in genetic risk and intervention outcomes may underlie GC by subtypes. GC of the intestinal type is known to develop through a cascade progression of multistage gastric lesions. In Linqu, the intestinal type of GC accounts for most cases. However, information on Lauren histological types was available only for limited SIT cases and unavailable for CKB. Further work is warranted to elucidate the identified genetic susceptibility for intestinal or diffuse type separately.

Second, although we made an attempt to leverage genetic predisposition to gastric lesion progression for GC risk and its primary prevention assessment, we had a modest sample size of trial participants, which precluded the possibility of conducting analyses in finer categories of genetic risk. An independent validation set for the association of chemoprevention with GC genetic risk was not available.

Third, we did not test *H pylori* infection during the follow-up of the SIT and cannot assess the long-term associations of 1-time *H pylori* treatment stratified by posttreatment infection in different genetic risk groups. Information on *H pylori* infection was not available for the CKB cohort.

Fourth, the past decades have seen changes in the procedures for endoscopic examinations and criteria for histopathologic diagnoses, which possibly raises concerns on the comparability of gastric lesions over time. Our team has placed particular emphasis on maintaining established standards, ensuring that all follow-up activities have adhered to the protocol. In addition, we only assessed the risk of GC for the analysis of 3 interventions.

Fifth, our study has an exploratory nature in applying the PRS for the assessment of prevention strategies’ effectiveness, and the concern of misclassification cannot be fully addressed. Despite our efforts to replicate the PRS for GC risk, the datasets were not fully independent. The case-control study design also has a lower evidence level than cohort studies. Trial participants were enrolled from the Linqu area, so any extrapolation to other populations should be cautious. Therefore, further replication based on independent cohort studies, particularly in low-risk settings, are warranted before the translation of findings. Studies to elucidate whether the identified loci based on SIT reflect intrinsic heterogeneity in genetic susceptibility of GC between populations from high- and low-risk areas are also warranted.

## Conclusions

This cohort study found that appropriate primary GC prevention may be associated with lower risk of GC among those with high genetic risk, suggesting that chemoprevention strategies should be tailored to genetic risk for effective GC prevention. Integrating host and genetic characteristics for fine risk stratification and, ideally, with tailored regimen selection, would be warranted in future endeavors for optimized primary prevention of GC.

## Notes

Supplement 1.
eMethods.

eReferences.
**eTable 1.** Number of Participants in Each Intervention Category as Randomly Assigned in 1995 and According to Genetic Risk in the Current Analysis of the Shandong Intervention Trial **eTable 2.** Characteristics of Study Participants in Different Datasets **eTable 3.** Gastric Histopathology in 1994, 1999, 2003, and 2022 According to the Gastric Histopathology in 1989 of the Shandong Intervention Trial Participants **eTable 4.** Association Results of 51 SNVs in Different Stages **eTable 5.** Analysis for Previously Reported Genome-Wide Significant SNVs in the GWAS-Catalog **eTable 6.** Sensitivity Analyses of 12 Gastric Cancer–Associated SNVs Based on the Shandong Intervention Trial **eTable 7.** Sensitivity Analysis of Polygenic Risk Scores Associated With the Risk of Gastric Cancer in the SIT, CKB, and External Case-Control Validation Set **eTable 8.** Sensitivity Analyses of *H pylori* Treatment and Nutrition Supplementation Associated With Incident Gastric Cancer by Different Genetic Risks Based on the Shandong Intervention Trial **eTable 9.** Differentially Expressed Gene(s) Mapped Within 1000 Kb of Each Genomic Locus in Stomach Tissues **eTable 10.** KEGG Network Enrichment Analysis for 123 Genes Expressed in Gastric Tissues With Potential Biological Significance **eTable 11.** Identification of Differentially Expressed Genes Between Advanced and Mild Gastric Lesions **eTable 12.** Cis-eQTL Analysis Based on Analysis of Linqu Stomach Tissue–Based Datasets **eTable 13.** Functional Annotation for rs5995654 and Its Nearby Variants **eTable 14.** Gene-Intervention Interaction Analysis for rs9607601 **eFigure 1.** Study Design **eFigure 2.** Quality Control for Selection of Genetic Variants **eFigure 3.** Flow Diagram of Functional Annotation **eFigure 4.** Gastric Histopathology of the Shandong Intervention Trial Participants **eFigure 5.** Q-Q Plot for Longitudinal Genome-Wide Association Analysis **eFigure 6.** Associations of Polygenic Risk Scores With the Risk of Incident Gastric Cancer **eFigure 7.** Restricted Cubic Spline Curves of Polygenic Risk Score and Incident Gastric Cancer Risk **eFigure 8.** Determination of PRS Cutoff Percentile for Genetic Risk Classification and the Association of *H pylori* Treatment With GC Incidence **eFigure 9.** Association of Nutrition Supplementation With Gastric Cancer for Low or High Genetic Risk Stratified by *H pylori* Infection **eFigure 10.** Schoenfeld Residuals Test for Using Fine-Gray Models **eFigure 11.** Joint Effect of *H pylori* Treatment and Vitamin Supplementation by Genetic Risk Among *H pylori*–Positive Participants **eFigure 12.** Regional Plots for the 12 Genomic Loci **eFigure 13.** Variant Prioritization **eFigure 14.** Roadmap Annotation of 22q13.1

Supplement 2.
Data Sharing Statement
