# Errors in Abstract, Figure 3, and Supplement 1

**DOI:** 10.1001/jamanetworkopen.2024.25166

**Published:** 2024-06-28

In the Original Investigation titled “*Helicobacter pylori* Treatment and Gastric Cancer Risk Among Individuals With High Genetic Risk for Gastric Cancer,”^1^ published on May 29, 2024, there were errors in the Abstract, Figure 3, and Supplement 1. In the Abstract, the hazard ratio for individuals with high genetic risk should have read 0.45 (95% CI, 0.24-0.82). In the caption for Figure 3, the sample size for gastric cancer tissue from The Cancer Genome Atlas-Stomach Adenocarcinoma should have read 431, and the sample size for independent nontumor tissue from Genotype-Tissue Expression should have read 174. This change was also made in the eMethods and eTable 9 in Supplement 1. This article has been corrected.^1^
