# Supplementary material for: Helicobacter pylori Treatment and Gastric Cancer Risk Among Individuals With High Genetic Risk for Gastric Cancer
Source: JAMA Netw Open. 2024 May 29;7(5):e2413708. doi: 10.1001/jamanetworkopen.2024.13708 (PMC11137637; doi:10.1001/jamanetworkopen.2024.13708)
Supplement: Supplement 1. — eMethods. eReferences. eTable 1. Number of Participants in Each Intervention Category as Randomly Assigned in 1995 and According to Genetic Risk in the Current Analysis of the Shandong Intervention Trial eTable 2. Characteristics of Study Participants in Different Datasets eTable 3. Gastric Histopathology in 1994, 1999, 2003, and 2022 According to the Gastric Histopathology in 1989 of the Shandong Intervention Trial Participants eTable 4. Association Results of 51 SNVs in Different Stages eTable 5. Analysis for Previously Reported Genome-Wide Significant SNVs in the GWAS-Catalog eTable 6. Sensitivity Analyses of 12 Gastric Cancer–Associated SNVs Based on the Shandong Intervention Trial eTable 7. Sensitivity Analysis of Polygenic Risk Scores Associated With the Risk of Gastric Cancer in the SIT, CKB, and External Case-Control Validation Set eTable 8. Sensitivity Analyses of H pylori Treatment and Nutrition Supplementation Associated With Incident Gastric Cancer by Different Genetic Risks Based on the Shandong Intervention Trial eTable 9. Differentially Expressed Gene(s) Mapped Within 1000 Kb of Each Genomic Locus in Stomach Tissues eTable 10. KEGG Network Enrichment Analysis for 123 Genes Expressed in Gastric Tissues With Potential Biological Significance eTable 11. Identification of Differentially Expressed Genes Between Advanced and Mild Gastric Lesions eTable 12. Cis-eQTL Analysis Based on Analysis of Linqu Stomach Tissue–Based Datasets eTable 13. Functional Annotation for rs5995654 and Its Nearby Variants eTable 14. Gene-Intervention Interaction Analysis for rs9607601 eFigure 1. Study Design eFigure 2. Quality Control for Selection of Genetic Variants eFigure 3. Flow Diagram of Functional Annotation eFigure 4. Gastric Histopathology of the Shandong Intervention Trial Participants eFigure 5. Q-Q Plot for Longitudinal Genome-Wide Association Analysis eFigure 6. Associations of Polygenic Risk Scores With the Risk of Incident Gastric Cancer eFigure 7. Restricted Cubi [file jamanetwopen-e2413708-s001.pdf]

Supplemental Online Content

Xu HM, Han Y, Liu ZC, et al. *Helicobacter pylori* Treatment and Gastric Cancer Risk Among Individuals With High Genetic Risk for Gastric Cancer. *JAMA Netw Open.* 2024;7(5):e2413708. doi:10.1001/jamanetworkopen.2024.13708

eMethods.

eReferences.

eTable 1. Number of Participants in Each Intervention Category as Randomly Assigned in 1995 and According to Genetic Risk in the Current Analysis of the Shandong Intervention Trial

eTable 2. Characteristics of Study Participants in Different Datasets

eTable 3. Gastric Histopathology in 1994, 1999, 2003, and 2022 According to the Gastric Histopathology in 1989 of the Shandong Intervention Trial Participants

eTable 4. Association Results of 51 SNVs in Different Stages

eTable 5. Analysis for Previously Reported Genome-Wide Significant SNVs in the GWAS-Catalog

eTable 6. Sensitivity Analyses of 12 Gastric Cancer–Associated SNVs Based on the Shandong Intervention Trial

eTable 7. Sensitivity Analysis of Polygenic Risk Scores Associated With the Risk of Gastric Cancer in the SIT, CKB, and External Case-Control Validation Set

eTable 8. Sensitivity Analyses of *H pylori* Treatment and Nutrition Supplementation Associated With Incident Gastric Cancer by Different Genetic Risks Based on the Shandong Intervention Trial

eTable 9. Differentially Expressed Gene(s) Mapped Within 1000 Kb of Each Genomic Locus in Stomach Tissues

eTable 10. KEGG Network Enrichment Analysis for 123 Genes Expressed in Gastric Tissues With Potential Biological Significance

eTable 11. Identification of Differentially Expressed Genes Between Advanced and Mild Gastric Lesions

eTable 12. Cis-eQTL Analysis Based on Analysis of Linqu Stomach Tissue–Based Datasets

eTable 13. Functional Annotation for rs5995654 and Its Nearby Variants

eTable 14. Gene-Intervention Interaction Analysis for rs9607601

eFigure 1. Study Design

eFigure 2. Quality Control for Selection Of Genetic Variants

eFigure 3. Flow Diagram of Functional Annotation

eFigure 4. Gastric Histopathology of the Shandong Intervention Trial Participants

eFigure 5. Q-Q Plot for Longitudinal Genome-Wide Association Analysis

eFigure 6. Associations of Polygenic Risk Scores With the Risk of Incident Gastric Cancer

© 2024 XU HM et al. *JAMA Network Open.*

eFigure 7. Restricted Cubic Spline Curves of Polygenic Risk Score and Incident Gastric Cancer Risk

eFigure 8. Determination of PRS Cutoff Percentile for Genetic Risk Classification and the Association of *H pylori* Treatment With GC Incidence

eFigure 9. Association of Nutrition Supplementation With Gastric Cancer for Low or High Genetic Risk Stratified by *H pylori* Infection

eFigure 10. Schoenfeld Residuals Test for Using Fine-Gray Models

eFigure 11. Joint Effect of *H pylori* Treatment and Vitamin Supplementation by Genetic Risk Among *H pylori*–Positive Participants

eFigure 12. Regional Plots for the 12 Genomic Loci

eFigure 13. Variant Prioritization

eFigure 14. Roadmap Annotation of 22q13.1

This supplemental material has been provided by the authors to give readers additional information about their work.

eMethods

Gastroscopy and histopathology for the Shandong Intervention Trial (SIT) participants

Scheduled endoscopies were performed in 1989, 1994, 1999 and 2003 for all participants (**eTable-3 and eFigure-4**). Besides, we had scheduled gastroscopies in 2007 for those with a diagnosis of moderate-to-severe dysplasia (DYS) at any biopsy site in 2003 and repeated gastroscopies every six months to one year after 2008 for those with a diagnosis of moderate-to-severe DYS at any biopsy site or with mild DYS at two or more sites in 2003. Appending histopathological diagnoses from scheduled endoscopies after 2003, cancer registry or autopsy report data were also incorporated for gastric cancer (GC) diagnoses. To avoid delayed reporting of new cancer cases to the registry system, a local physician supervised active clinical follow-up in each village and documented cancer occurrences. Peking University Cancer Hospital & Institute staff members visited each township hospital quarterly to gather information on cancers, and visited villages every 6 months to validate new cancer events. Reported new GC cases were confirmed through pathological review. Integrating these data sources, the fifth-time histopathological diagnosis (2022) was assigned. Study participants had excellent compliance with follow-up, with only 2·3% missing one or more scheduled endoscopies. Other than recorded deaths, none was lost to follow-up for cancer occurrence among participants of this study.

All endoscopic examinations were conducted by a panel of experienced gastroenterologists, with biopsies taken from seven standard mucosal sites for all participants and reviewed blindly by a team of pathologists following the criteria proposed by the Chinese Association of Gastric Cancer and Updated Sydney System[1-4]. Dr. Ji-You Li, Peking University Cancer Hospital, supervised the pathological

© 2024 XU HM et al. *JAMA Network Open.*

diagnoses of SIT until his retirement in 2012. Prior quality control assessment revealed satisfying concordance between the histopathologic diagnoses of Dr. Ji-You Li and Dr. Pelayo Correa (Vanderbilt University)[5]. Dr. Zhong-Wu Li (Peking University Cancer Hospital, trained by Dr. Ji-You Li as his mentor) supervised the review of the histopathologic diagnoses of slides since 2012. The team has adhered to resilient and relatively consistent criteria during follow-up to ensure the comparability of gastric lesions over time. Each participant was given a global diagnosis based on the most severe histology among all biopsies of each endoscopic examination. A global severity score was assigned according to histopathologic diagnosis, with 0-9 for normal, superficial gastritis (SG), mild/moderate chronic atrophic gastritis (CAG), severe CAG, superficial intestinal metaplasia (IM), deep IM, mild DYS, moderate DYS, severe DYS and GC respectively.

Although the name of SIT was not officially designated until the real trial started in 1995, we refer to participants of the whole study period (1989-2022) as the 'SIT cohort' for convenience.

CKB Cohort follow-up

During the follow-up period up to December 31st, 2018, diseases, hospitalization events, and deaths were documented by linkage to the Chinese national health insurance database and local disease registry and death report systems. Annual active follow-up was performed to collect disease and vital status for those who failed to be linked to the health insurance database. Events with the International Classification of Diseases, 10th revision code of C16 were defined as developing gastric cancer during the follow-up. As the cancer registry was only obliged to report to the level of code 8140 (e.g. M-8140/3 1) for all gastric adenocarcinomas, information on Lauren

© 2024 XU HM et al. *JAMA Network Open.*

subtype is yet unavailable. In addition to cancer registry, the CKB also collected further clinical information of gastric cancer via a Portable Verification Device (PVD) system by reviewing the medical records at hospital for a proportion of cases. The available data from reviewing medical records reported an accuracy of 91% for GC diagnoses, among which 85% were of adenocarcinoma subtype. Efforts are ongoing within the framework of PVD program to collect further specific information as well as a copy of pathology report image, in which some may indicate the specific pathology subtype, e.g. signet ring cell tumor, etc, and also help clarify other classifications (e.g. Lauren) in the future.

Case-control validation set

Genetic data of a case-control study, including 702 GC cases and 692 controls from Linqu county, was used as an external validation set for polygenetic risk score (PRS) associated with GC risk. This ongoing case-control study was designed to examine the multiomics signatures for GC, including germline genetic polymorphisms, *H.pylori* serotypes and other omics data, with genotyping of peripheral blood leukocyte DNA samples completed recently. For this purpose, we enrolled invasive primary GC cases and non-GC controls aged 30-75 years. All invasive GC cases (the International Classification of Diseases code of C16, 10th revision) were diagnosed between Jan 2011 and Dec 2022. Controls were selected in 2023 from Linqu residents attending physical examination and upper gastrointestinal cancer screening. Information on age and sex was documented utilizing the identity card of China. We excluded those refusing to provide informed consent, ages outside the range of 30-75 years, those with previous diagnosis of cancer (other than the current diagnosis of invasive GC for cases group, except nonmelanoma skin cancer), peptic ulcer, bleeding

© 2024 XU HM et al. *JAMA Network Open.*

disorder, heart failure, renal disorder, liver disease, emphysema, mental or psychiatric illness, or other serious medical conditions, receiving previous *H.pylori* treatment, or refusing to attend the CUBT test for detection of *H.pylori* infection status. For the current analysis, among 1430 subjects (717 cases and 713 controls) with DNA samples meeting the requirement of genotyping platform, 1394 (702 cases and 692 controls) had genotype data passing quality control (**eFigure-2**) for the subsequent analysis.

Genotyping and imputation for SIT participants and the external case-control validation set

Peripheral blood leukocyte DNA samples were genotyped using the Global Screening Array beadchip (Illumina. lnc) for SIT participants and Asian Screening Array beadchip (Illumina. lnc) for the external case-control validation set. Genotyped variants were excluded if they had a call rate <95%, a *P*-value for Hardy–Weinberg Equilibrium (HWE) $<1·0\times10^{-6}$ or a minor allele frequency (MAF) ≤0·5% or were duplicated variants. Samples were removed if they had abnormal missing rate of variants (>5%) or heterozygosity rate (>±3 standard deviation(SD) from the mean), sex discrepancy, and were duplicated samples, which followed standard procedures of GWAS quality control[6].

For imputation of SNPs, we used SHAPEIT (v2) to phase qualified genotypes for each chromosome[7]. Imputation was performed for each 5-Megabase (Mb) interval with IMPUTE2 (v. 2·3·1) using a merged reference panel from 1000 Genomes Project (the Phase III integrated variant set release, across 2,504 samples)[8]. Imputed variants were excluded if they had an imputation quality score INFO≤0·3, a MAF≤0·5%, or a missing rate≥10% (**eFigure-2**).

© 2024 XU HM et al. *JAMA Network Open.*

Genotyping and imputation for the China Kadoorie Biobank (CKB)

Custom-designed Affymetrix Axiom arrays (BGI Group, Shenzhen, China) were used for CKB. Samples with call rate ≤95% or sex discrepancy, with extreme heterozygosity (>±3 SD from the mean), or with extreme homozygosity rate (>±3 SD from the mean) were excluded. Variants with call rate >0·98, plate effect $P > 10^{-6}$, batch effect $P > 10^{-6}$, HWE deviations $P > 10^{-6}$ (combined 10 degrees of freedom Chi-squared test from 10 regions) and MAF difference from 1000 Genomes East Asian frequencies <0·2 were identified. The qualified genotypes for each chromosome were phased with SHAPEIT and imputation was performed using the same method with the SIT. Variants with imputation quality score INFO>0·3 or MAF>0·5% were included (**eFigure-2**). Mismatching alleles between SIT and CKB were checked and resolved by strand-flipping or recoding.

Statistical analysis

Based on multiple-time histopathological diagnoses of gastric lesions in the SIT, longitudinal GWAS was conducted using a generalized linear mixed model (GLMM, MAGEE package in R, version 4·2·2)[9], with the formula as follows:

$$Global\ severity\ score = \beta_{0j} + \beta_{1j}Time + e_{ij}$$

Where

$$\beta_{0j} = \gamma_{00} + \gamma_{01}SNP + \gamma_{02}Age + \gamma_{03}Sex + \gamma_{04}H.pylori + \gamma_{05}PC1 + \cdots + \gamma_{09}PC5 + \mu_{oj}$$

and

$$\beta_{1j} = \gamma_{10} + \gamma_{11}SNP + \mu_{1j}$$

The GLMM model, containing fixed effect and random effect modules, deals with

© 2024 XU HM et al. *JAMA Network Open.*

longitudinal measures by allowing each person i to have a model with time point *j,* which is more convergent than traditional case-control based-GWAS analytical models. The time point *j* indicates the time of scheduled endoscopy. The intercept μ_{oj} is assumed to account for random individual effects for a n×n empirical genetic kinship matrices V_k, and for random individual effects of the intercept not attributable to genetic relatedness. In addition, μ_{1j} represents the random effects of the time slope and e_{ij} is a matrix of random errors. The fixed effects include an intercept, SNP (genetic dosage), time, SNP×Time interaction, and covariates including age (time-varying), sex, *Helicobacter pylori* infection, and five principal components (PCs) of ancestry accounting for population stratification. Multiple imputation was used for missing data of histopathological diagnoses[10]. Multiple imputation was not conducted for decreased individuals, who were then assigned missing values of histopathological diagnoses for the subsequent time points. The major effect estimate of interest was γ11 for the interaction (SNP×Time) variable, which illustrates how genetic variants affect the progression of gastric lesions over time[11]. SNPs with $P<5\times10^{-4}$ for the interaction term were examined for the risk of incident GC based on the CKB set-1 using SAIGE[12].

The standard C+T (clumping + thresholding) method was used to identify the independent genetic effects for GC. To address the issue of linkage disequilibrium (LD) between nearby variants, a clumping procedure was employed by grouping SNPs in LD ($r^2>0.2$) with each other. Through this process, we selected the lead (index) SNPs, specifically the most significant SNP associated with GC within each LD block. By retaining these SNPs, we ensured that they were largely independent of each other, enabling us to sum their effects accurately. To comprehensively assess individual's genetic predisposition to GC, we included the lead SNPs that were

associated with progression of gastric lesions ($P<5\times10^{-4}$) in the SIT cohort and consistently associated with incident GC in CKB set-1 ($P<0.05$) for the construction of the polygenic risk score (PRS). For each individual, we weighted the dosage of the effect allele for each SNP by its respective effect estimate (β) using PLINK v1·9.

The Fine-Gray models accounting for death from causes other than GC as competing risk were used to calculate the hazard ratios (HRs) and 95% confidence intervals (CI) for the association between normalized PRSs and incident GC risk based on the CKB set-2, with follow-up person-years as the time axis. We tested the proportional subdistribution hazard assumption utilizing Schoenfeld residuals methods. The association of PRS with GC risk was further tested in the CKB set-2. Potential non-linear association was assessed using restricted cubic spline analysis.

To evaluate the performance of the PRS for GC risk discrimination, the concordance index (C-index) was used based on recommendations by an expert consensus panel for improving reporting standards for polygenic risk score[13]. C-index for the PRS in classifying GC risk was calculated based on a gradient boosting method and its 95% CI was derived by bootstrapping in CKB set-1. For the analyses, the PRS (a continuous variable) was incorporated which, as per convention, undergoes mean standardization, and other covariates, including age, sex, and PCs were adjusted for. The performance was then evaluated in CKB-set 2 and SIT cohort respectively with C-index calculated.

To further validate the performance of PRS, an external case-control dataset based on Linqu residents was utilized to examine the continuous PRS and deciles of PRS associated with GC. Logistic regression models were used for the analysis, adjusting for age, sex, *H. pylori* infection and PCs. C-index for discriminating GC risk was also calculated.

© 2024 XU HM et al. *JAMA Network Open.*

We rigorously determined the PRS cut-off value, aiming to differentiate individuals into low or high genetic risk categories through structured machine learning workflows. We utilized the gradient boosting method to identify the most appropriate PRS cut-off threshold, methodically assessing values incremented by 5%, spanning from 50% to 95%. The threshold that manifested the highest C-index value was subsequently adopted as the benchmark PRS cut-off for ensuing analytical procedures. In our study, the 75th percentile of PRS with the highest C-index was deemed as the most appropriate cut-off value for classifying genetic risk. We therefore defined individuals with the top quartile of PRS as having a high genetic-risk for GC and otherwise having a low genetic-risk.

We applied the PRS to SIT trial participants and compared the successful eradication rate of *H. pylori* between high (top quartile of PRS) and low genetic-risk individuals using chi-squared test. Based on the intention-to-treat approach, the effect of *H. pylori* treatment, vitamin supplementation, and garlic supplementation was evaluated for SIT participants with a high and low genetic risk for GC and three sets of analyses were conducted. First, we examined the effect of each intervention (active treatment vs. placebo) for overall trial participants. Second, the effect of vitamin supplementation and garlic supplementation for high and low genetic-risk individuals was assessed separately for baseline *H. pylori* positive and negative individuals. Third, as we previously reported the protective effect of *H. pylori* treatment and vitamin supplementation against GC risk, we further evaluated the joint effect of receiving different combinations of these two interventions (placebo/placebo, *H. pylori* treatment only, vitamin supplementation only, and both active treatments). For each analysis, *P*-values for interaction (*P*-interaction) between the PRS and assessed intervention were calculated. The absolute risk reduction (ARR) was calculated as the

difference in GC incidence between comparison groups over 27·1-year's follow-up[14]. The number of participants needed to treat (NNT) to prevent one GC in 27·1 years was also calculated[14,15]. Analyses were conducted using R (version 4·2.2).

Examination of GWAS-Catalog reported variants for gastric cancer (GC) of Asian

We sought to examine whether GWAS-Catalog (https://www.ebi.ac.uk/gwas/) summarized variants for GC of Asian were associated with the risk of gastric lesion progression using GLMM, with 50 of 67 genotyped/imputed and passing quality control in the SIT. Analyses were conducted to examine their associations for gastric lesion progression (SIT) and GC risk (CKB) separately. Variants with nominal significance (P<0·05) and consistent direction of effect associated with GC were highlighted for further analyses.

Sensitivity analysis

While the primary GLMM analysis utilized the histopathological diagnoses of all five times, a sensitivity analysis was also conducted by having four-time diagnoses (1994, 1999, 2003, 2022) during follow-up as the outcome variable in the longitudinal matrix of GLMMs and adjusting for baseline diagnosis (1989) as a covariate, along with other covariates as described for the primary analysis. Another sensitivity analysis was also conducted by additionally adjusting for three chemopreventions, including *H.pylori* treatment (without attending the intervention trial in 1995, *H.pylori* negative, *H.pylori* positive receiving active treatment, or receiving placebo), vitamin supplementation (without attending the intervention trial, receiving active treatment, or receiving placebo) and garlic supplementation (without attending the intervention trial, receiving active treatment, or receiving placebo), along with age, sex, and five

© 2024 XU HM et al. *JAMA Network Open.*

PCs as covariates. To ease the translation of PRS models, a sensitivity analysis on the association of PRS with GC was also conducted without adjusting for PCs.

For the analysis on the intervention effect by genetic risk, we also performed sensitivity analyses to test the robustness of polygenic risk score (PRS) modifying the intervention effects by defining individuals with the PRS above the threshold incremented by 5%, spanning from 50% to 95%, as having high genetic-risk respectively, or excluding three SNPs that had MAF≤2% in the SIT cohort (rs78078728, rs10147214, and rs1110549) or removing the PCs from the adjustment for covariates.

Functional annotation

The fine-mapping and functional annotation procedure is described in **eFigure-3**. FUMA v.1·3.6a[16] was used to functionally annotate and map variants associated with the risk of gastric lesion progression and GC. We defined each genomic locus for independent significant SNPs by incorporating variants located within 250 kilobases (Kb) and with $r^2>0·4$ referred by 1000 Genome Asian reference information[8]. Functional annotation of each SNP was acquired from ANNOVAR cytoband database[17]. Regional plots were generated using LocusZoom (https://my.locuszoom.org/).

The individual genomic risk loci were mapped to genes using FUMA v.1.3.6a[16]. Genes located within 1 Mb of the genomic locus were defined as positional mapped genes. Among them, analyses were then conducted to identify genes expressed in gastric tissues, differentially expressed genes (DEGs) between tumor and non-tumor gastric tissues, and expression quantitative trait loci (eQTL). First, our in-house RNA sequencing data (BGI's DNBSEQ platform) of 88 non-tumor stomach tissues

collected in Linqu were utilized to identify genes expressed in gastric tissues, defined as genes with a transcript per million (TPM) >1 in more than 50% of these given samples[18]. KEGG pathway enrichment analysis was performed for the identified genes in gastric tissues for interpretation of biological insights[19]. Second, to discover differentially expressed genes (DEGs) between GC tumor and non-tumor tissues, RNA-seq-based gene expression in stomach tumor tissue (TCGA-STAD[20], n=413) was compared with adjacent non-tumor tissue (n=36) and normal stomach tissue (GTEx[21], n=174) using Limma package in R. DEGs were defined as genes with $|\log_2\text{FoldChange}\ (\log_2\text{FC})|>2.64$ (mean±2 SD of $\log_2$FC), and Bonferroni-adjusted $P<4.07\times10^{-4}$ (0·05/123 genes expressed in gastric tissues). The DEGs were further examined for expressions between individuals with mild gastric lesions (SG/CAG, n=54) and advanced gastric lesions (IM/DYS, n=34) based on our RNA-seq dataset in Linqu. Third, to assess the impact of genetic variants on cis-expression of highlighted DEGs, eQTL analysis was performed for genes located within 1 Mb up- or downstream of candidate SNPs based on the Linqu RNA-seq data of non-tumor stomach tissues (n=65 of 88 with genotypic data). MatrixEQTL (R package) was used for the linear regression analysis adjusting for age and sex.

For stomach-specific cis-eQTLs based on Linqu RNA-seq data, the associations with cis-expression levels in the blood were checked based on data of eQTLGen consortium[22]. In addition to these two terms on stomach-based and blood-based eQTLs, variant prioritization was performed incorporating another 5 functional annotation terms via HaploReg[23] (v4·1, Haploreg Promoter Histone Marks, Haploreg Enhancer Histone Marks, Haploreg DNase, Haploreg Proteins bound, Haploreg Motifs changed), and 3 terms via Regulome DB[24] (v2·1, RegulomeDB eQTLs, TF binding, DNase peak). Prioritization score integrating these ten terms was calculated

for the candidate variants (within±250Kb and $r^2>0{\cdot}4$ of independent variant), with a score of 1 assigned for each term. Variant with a high prioritization score would be deemed with strong supporting evidence for biological relevance. For visualization purposes, we utilized NIH Roadmap human reference epigenome[25] to acquire enrichment of DNase I hypersensitivity site (DNase) and histone marks in the genomic locus. Two categories in the Roadmap database (E092 Fetal stomach and E110 Stomach of Mucosa) were chosen to represent epigenomes on stomach tissues.

Reference

1. Li WQ, Zhang JY, Ma JL, et al. Effects of Helicobacter pylori treatment and vitamin and garlic supplementation on gastric cancer incidence and mortality: follow-up of a randomized intervention trial. *BMJ.* 2019;366:l5016.
2. You WC, Blot WJ, Li JY, et al. Precancerous gastric lesions in a population at high risk of stomach cancer. *Cancer research.* 1993;53(6):1317-1321.
3. You W-c, Brown LM, Zhang L, et al. Randomized double-blind factorial trial of three treatments to reduce the prevalence of precancerous gastric lesions. *Journal of the National Cancer Institute.* 2006;98(14):974-983.
4. Dixon MF, Genta RM, Yardley JH, Correa P. Classification and grading of gastritis. The updated Sydney System. International Workshop on the Histopathology of Gastritis, Houston 1994. *The American journal of surgical pathology.* 1996;20(10):1161-1181.
5. You W-c, Brown LM, Zhang L, et al. Randomized Double-Blind Factorial Trial of Three Treatments To Reduce the Prevalence of Precancerous Gastric Lesions. *JNCI: Journal of the National Cancer Institute.* 2006;98(14):974-983.
6. Anderson CA, Pettersson FH, Clarke GM, Cardon LR, Morris AP, Zondervan KT. Data quality control in genetic case-control association studies. *Nat Protoc.* 2010;5(9):1564-1573.
7. Howie BN, Donnelly P, Marchini J. A flexible and accurate genotype imputation method for the next generation of genome-wide association studies. *PLoS Genet.* 2009;5(6):e1000529.
8. Auton A, Brooks LD, Durbin RM, et al. A global reference for human genetic variation. *Nature.* 2015;526(7571):68-74.

© 2024 XU HM et al. *JAMA Network Open.*

9. Wang X, Lim E, Liu CT, et al. Efficient gene-environment interaction tests for large biobank-scale sequencing studies. *Genetic epidemiology.* 2020;44(8):908-923.
10. Spratt M, Carpenter J, Sterne JAC, et al. Strategies for Multiple Imputation in Longitudinal Studies. *American Journal of Epidemiology.* 2010;172(4):478-487.
11. Allen RJ, Oldham JM, Jenkins DA, et al. Longitudinal lung function and gas transfer in individuals with idiopathic pulmonary fibrosis: a genome-wide association study. *The Lancet Respiratory medicine.* 2023;11(1):65-73.
12. Zhou W, Nielsen JB, Fritsche LG, et al. Efficiently controlling for case-control imbalance and sample relatedness in large-scale genetic association studies. *Nature genetics.* 2018;50(9):1335-1341.
13. Wand H, Lambert SA, Tamburro C, et al. Improving reporting standards for polygenic scores in risk prediction studies. *Nature.* 2021;591(7849):211-219.
14. Nuovo J, Melnikow J, Chang D. Reporting number needed to treat and absolute risk reduction in randomized controlled trials. *Jama.* 2002;287(21):2813-2814.
15. Cook RJ, Sackett DL. The number needed to treat: a clinically useful measure of treatment effect. *BMJ (Clinical research ed).* 1995;310(6977):452-454.
16. Watanabe K, Taskesen E, van Bochoven A, Posthuma D. Functional mapping and annotation of genetic associations with FUMA. *Nature communications.* 2017;8(1):1826.
17. Wang K, Li M, Hakonarson H. ANNOVAR: functional annotation of genetic variants from high-throughput sequencing data. *Nucleic acids research.* 2010;38(16):e164.

© 2024 XU HM et al. *JAMA Network Open.*

18. Wang C, Dai J, Qin N, et al. Analyses of rare predisposing variants of lung cancer in 6,004 whole genomes in Chinese. *Cancer cell.* 2022;40(10):1223-1239.e1226.
19. Kanehisa M, Araki M, Goto S, et al. KEGG for linking genomes to life and the environment. *Nucleic acids research.* 2007;36(suppl_1):D480-D484.
20. Tomczak K, Czerwińska P, Wiznerowicz M. The Cancer Genome Atlas (TCGA): an immeasurable source of knowledge. *Contemporary oncology (Poznan, Poland).* 2015;19(1a):A68-77.
21. The Genotype-Tissue Expression (GTEx) project. *Nature genetics.* 2013;45(6):580-585.
22. Võsa U, Claringbould A, Westra H-J, et al. Large-scale cis-and trans-eQTL analyses identify thousands of genetic loci and polygenic scores that regulate blood gene expression. *Nature genetics.* 2021;53(9):1300-1310.
23. Ward LD, Kellis M. HaploReg v4: systematic mining of putative causal variants, cell types, regulators and target genes for human complex traits and disease. *Nucleic acids research.* 2016;44(D1):D877-881.
24. Boyle AP, Hong EL, Hariharan M, et al. Annotation of functional variation in personal genomes using RegulomeDB. *Genome research.* 2012;22(9):1790-1797.
25. Roadmap EC, Kundaje A, Meuleman W, et al. Integrative analysis of 111 reference human epigenomes. *Nature.* 2015;518(7539):317-330.

© 2024 XU HM et al. *JAMA Network Open.*

eTable 1. Number of participants in each intervention category as randomly assigned in 1995 and according to genetic risk in the current analysis of the Shandong Intervention Trial.

Intervention			No. of subjects			
H.pylori treatment	Garlic supplementation	Vitamin supplementation	Total subjects attending the trial	Subjects attending the trial with qualified genotype data	Low genetic risk	High genetic risk
***H. pylori* seropositive subjects in 1994**						
A	A	A	283	226	166	60
A	A	P	283	223	165	58
A	P	A	281	223	168	55
A	P	P	283	229	169	60
P	A	A	279	229	165	64
P	A	P	282	233	169	64
P	P	A	283	235	173	62
P	P	P	284	255	187	68
Total			2258	1853	1362	491
***H. pylori* seronegative subjects in 1994**						
P	A	A	274	185	149	36
P	A	P	277	185	150	35
P	P	A	277	186	134	52
P	P	P	279	195	146	49
Total			1107	751	579	172

We defined individuals with the genetic risk score at the top quartile (25%) as having a high genetic risk, otherwise having a low genetic risk.
Abbreviations: A, active treatment; *H. pylori*, *Helicobacter pylori*; P, Placebo.

© 2024 XU HM et al. *JAMA Network Open.*

eTable 2. Characteristics of study subjects in different datasets.

	SIT	CKB [a]			External case-control validation set
		set-1	set-2	Total	
Age, years (±SD)	46.95±9.12	53.74±11.00	53.64±11.01	53.69±11.00	54.54±7.66
Sex					
Male	1,387 (49.25)	21,339 (42.75)	21,532 (42.79)	42,871 (42.77)	867 (62.20)
Female	1,429 (50.75)	28,573 (57.25)	28,784 (57.21)	57,357 (57.23)	527 (37.80)
Region					
Linqu	2,816 (100.00)	N.A.	N.A.	N.A.	1,394 (100.00)
Qingdao	N.A.	4,119 (8.25)	4,087 (8.12)	8,206 (8.19)	N.A.
Harbin	N.A.	6,493 (13.01)	6,557 (13.03)	13,050 (13.02)	N.A.
Haikou	N.A.	2,840 (5.69)	2,938 (5.84)	5,778 (5.76)	N.A.
Suzhou	N.A.	3,946 (7.91)	4,001 (7.95)	7,947 (7.93)	N.A.
Liuzhou	N.A.	4,347 (8.71)	4,389 (8.72)	8,736 (8.71)	N.A.
Chengdu	N.A.	5,286 (10.59)	5,335 (10.60)	10,621 (10.60)	N.A.
Tianshui	N.A.	5,028 (10.07)	5,022 (9.98)	10,050 (10.03)	N.A.
Xinxiang	N.A.	5,668 (11.36)	5,688 (11.30)	11,356 (11.33)	N.A.
Jiaxing	N.A.	5,926 (11.87)	6,089 (12.10)	12,015 (11.99)	N.A.
Changsha	N.A.	6,259 (12.54)	6,210 (12.34)	12,469 (12.44)	N.A.
Total participants	2,816 (100.00)	49,912 (100.00)	50,316 (100.00)	100,228 (100.00)	1,394 (100.00)
Number of gastric cancer	147	375	450	825	702

[a] Subjects in the CKB cohort were randomly and equally divided into set-1 (for identifying gastric cancer-related SNPs) and set-2 (for assessing the risk of gastric cancer associated with the integrated polygenic risk score).
Abbreviations: CKB, China Kadoorie Biobank; N.A., not applicable; SIT, Shandong Intervention Trial

© 2024 XU HM et al. *JAMA Network Open.*

eTable 3. Gastric histopathology in 1994, 1999, 2003 and 2022 according to the gastric histopathology in 1989 of the Shandong Intervention Trial participants.

	Histopathology in 1989									
	Normal	SG	Mild/ moderate CAG	Severe CAG	Superficial IM	Deep IM	Mild DYS	Moderate DYS	Severe DYS	Total
Histopathology in 1994										
Normal	0	1	2	2	0	1	0	0	0	6
SG	0	3	41	2	1	3	2	0	0	52
Mild/moderate CAG	0	34	761	105	95	126	65	4	0	1190
Severe CAG	0	1	60	21	15	16	7	1	0	121
Superficial IM	0	1	87	31	31	66	48	3	0	267
Deep IM	0	3	200	56	102	325	235	22	0	943
Mild DYS	0	1	26	10	15	69	80	12	3	216
Moderate DYS	0	0	2	2	0	3	4	0	1	12
Severe DYS	0	0	1	0	0	0	2	0	1	4
GC	0	0	2	0	0	3	0	0	0	5
Total	0	44	1182	229	259	612	443	42	5	2816
Histopathology in 1999										
Normal	0	0	0	0	0	0	0	0	0	0
SG	0	0	14	0	1	3	0	0	0	18
Mild/moderate CAG	0	22	576	67	60	79	44	4	1	853
Severe CAG	0	12	195	34	26	32	30	1	0	330
Superficial IM	0	4	96	23	22	44	18	1	0	208
Deep IM	0	5	239	77	109	329	221	19	0	999
Mild DYS	0	1	51	25	33	110	111	17	4	352
Moderate DYS	0	0	0	0	2	3	4	0	0	9
Severe DYS	0	0	0	0	1	0	2	0	0	3
GC	0	0	3	0	2	5	10	0	0	20
Total	0	44	1174	226	256	605	440	42	5	2792
Histopathology in 2003										
Normal	0	0	0	0	0	0	0	0	0	0
SG	0	1	95	5	8	10	6	0	0	125
Mild/moderate CAG	0	25	549	66	54	68	24	3	0	789
Severe CAG	0	2	46	4	4	4	4	0	0	64
Superficial IM	0	1	16	6	2	7	2	0	0	34
Deep IM	0	8	265	77	97	242	176	9	1	875
Mild DYS	0	5	159	61	77	236	185	21	2	746
Moderate DYS	0	0	10	3	6	9	9	4	1	42
Severe DYS	0	0	0	0	1	2	2	0	0	5
GC	0	0	0	1	1	7	9	3	1	22
Total	0	42	1140	223	250	585	417	40	5	2702
Histopathology in 2022										
Normal	0	0	0	0	0	0	0	0	0	0
SG	0	1	79	4	6	8	6	0	0	104
Mild/moderate CAG	0	22	456	48	44	56	18	2	0	646
Severe CAG	0	2	41	4	2	4	2	0	0	55

© 2024 XU HM et al. *JAMA Network Open.*

	Histopathology in 1989									
	Normal	SG	Mild/ moderate CAG	Severe CAG	Superficial IM	Deep IM	Mild DYS	Moderate DYS	Severe DYS	Total
Superficial IM	0	0	15	6	2	6	2	0	0	31
Deep IM	0	5	203	60	71	171	119	6	1	636
Mild DYS	0	5	126	44	57	161	122	13	1	529
Moderate DYS	0	0	7	3	4	4	7	3	1	29
Severe DYS	0	0	0	0	1	2	2	0	0	5
GC	0	0	23	5	8	32	29	3	0	100
Total	0	35	950	174	195	444	307	27	3	2135

Abbreviations: CAG, chronic atrophic gastritis; DYS, dysplasia; GC, gastric cancer; IM, intestinal metaplasia; SG, superficial gastritis.

© 2024 XU HM et al. *JAMA Network Open.*

eTable 4. Association results of 51 SNPs in different stages.

Chr:Position[a]	rsID	ANNOVAR annotation	Closest gene(s)[b]	Effect allele	SIT[c]				CKB[c]									
									EAF	set-1			set-2			Total		
					EAF	Beta	SE	*P*		Beta	SE	*P*	Beta	SE	*P*	Beta	SE	*P*
1:82227856	rs12066641	intronic	*ADGRL2*	A	0.06	0.10	0.03	1.12×10^{-4}	0.07	0.35	0.15	0.02	-0.02	0.14	0.87	0.14	0.10	0.17
1:82228979	rs12403675	intronic	*ADGRL2*	T	0.06	0.10	0.03	1.12×10^{-4}	0.07	0.35	0.15	0.02	-0.02	0.14	0.87	0.14	0.10	0.17
1:82232668	rs17107162	intronic	*ADGRL2*	T	0.06	0.10	0.03	1.20×10^{-4}	0.07	0.35	0.15	0.02	-0.02	0.14	0.87	0.14	0.10	0.16
**1:82242667**	**rs12070840**	**intronic**	***ADGRL2***	**T**	**0.06**	**0.09**	**0.02**	$\mathbf{2.40\times10^{-4}}$	**0.07**	**0.36**	**0.15**	**0.02**	**-0.02**	**0.14**	**0.89**	**0.15**	**0.10**	**0.15**
1:82243083	rs12072054	intronic	*ADGRL2*	G	0.06	0.09	0.02	1.71×10^{-4}	0.07	0.32	0.14	0.03	-0.02	0.13	0.86	0.13	0.10	0.18
1:82243449	rs11163366	intronic	*ADGRL2*	A	0.06	0.09	0.02	1.71×10^{-4}	0.07	0.32	0.15	0.03	-0.02	0.13	0.86	0.13	0.10	0.19
1:82243458	rs11163367	intronic	*ADGRL2*	T	0.06	0.09	0.02	1.71×10^{-4}	0.07	0.32	0.15	0.03	-0.02	0.13	0.86	0.13	0.10	0.19
**1:103580618**	**rs78078728**	**intergenic**	***COL11A1; LOC101928436***	**T**	**0.01**	**-0.26**	**0.06**	$\mathbf{3.91\times10^{-5}}$	**0.01**	**-0.47**	**0.24**	**0.050**	**-0.32**	**0.22**	**0.14**	**-0.40**	**0.16**	**0.01**
3:66090476	rs75874606	intergenic	*MAGI1;SLC25A26*	G	0.07	0.08	0.02	2.23×10^{-4}	0.07	0.30	0.15	0.05	-0.01	0.14	0.93	0.14	0.10	0.19
4:55941418	rs73818546	intergenic	*KIT;KDR*	A	0.05	0.12	0.03	5.22×10^{-6}	0.06	0.37	0.15	0.02	-0.17	0.14	0.21	0.08	0.10	0.45
4:55942323	rs7659911	intergenic	*KIT;KDR*	C	0.05	0.11	0.03	1.38×10^{-5}	0.06	0.36	0.15	0.02	-0.15	0.14	0.27	0.08	0.10	0.41
**4:55944618**	**rs41481345**	**downstream**	***KDR***	**G**	**0.05**	**0.10**	**0.03**	$\mathbf{5.82\times10^{-5}}$	**0.06**	**0.39**	**0.16**	**0.01**	**-0.15**	**0.14**	**0.29**	**0.10**	**0.11**	**0.36**
**4:130720635**	**rs2391536**	**ncRNA_intronic**	***LINC02465***	**G**	**0.95**	**-0.08**	**0.03**	$\mathbf{3.91\times10^{-4}}$	**0.93**	**-0.34**	**0.16**	**0.03**	**-0.03**	**0.14**	**0.82**	**-0.17**	**0.11**	**0.11**
5:116053615	rs10035198	intergenic	*SEMA6A-AS2;LINC02214*	G	0.02	-0.17	0.04	6.63×10^{-5}	0.03	-0.49	0.23	0.04	-0.06	0.22	0.77	-0.26	0.16	0.10
5:116056920	rs2087872	intergenic	*SEMA6A-AS2;LINC02214*	A	0.02	-0.17	0.04	6.05×10^{-5}	0.03	-0.50	0.23	0.04	-0.07	0.22	0.75	-0.26	0.16	0.10
**5:169093410**	**rs6879467**	**intronic**	***DOCK2***	**G**	**0.53**	**-0.06**	**0.01**	$\mathbf{1.71\times10^{-6}}$	**0.53**	**-0.17**	**0.08**	**0.03**	**-0.04**	**0.07**	**0.54**	**-0.10**	**0.05**	**0.04**
5:169096002	rs12657748	intronic	*DOCK2*	C	0.53	-0.06	0.01	1.41×10^{-6}	0.54	-0.16	0.08	0.03	-0.04	0.07	0.52	-0.10	0.05	0.05
5:169096774	rs7721990	intronic	*DOCK2*	C	0.53	-0.06	0.01	1.84×10^{-6}	0.54	-0.15	0.07	0.04	-0.04	0.07	0.60	-0.09	0.05	0.07

© 2024 XU HM et al. *JAMA Network Open.*

Chr:Position[a]	rsID	ANNOVAR annotation	Closest gene(s)[b]	Effect allele	SIT[c]				CKB[c]									
									EAF	set-1			set-2			Total		
					EAF	Beta	SE	*P*		Beta	SE	*P*	Beta	SE	*P*	Beta	SE	*P*
5:169097940	rs2306565	intronic	*DOCK2*	A	0.53	-0.06	0.01	2.24×10^{-6}	0.53	-0.16	0.07	0.04	-0.04	0.07	0.59	-0.09	0.05	0.06
5:169106370	rs7705072	intronic	*DOCK2*	G	0.53	-0.06	0.01	8.71×10^{-7}	0.53	-0.15	0.08	0.05	-0.02	0.07	0.76	-0.08	0.05	0.10
5:169109140	rs9313467	intronic	*DOCK2*	G	0.53	-0.06	0.01	1.61×10^{-6}	0.54	-0.15	0.07	0.04	-0.03	0.07	0.69	-0.09	0.05	0.08
6:78167122	rs7454412	intergenic	*LINC02540;HTR1B*	C	0.11	-0.09	0.02	7.23×10^{-6}	0.14	-0.25	0.11	0.03	0.02	0.10	0.86	-0.10	0.08	0.18
**6:151236016**	**rs9478852**	**intronic**	***MTHFD1L***	**G**	**0.02**	**0.14**	**0.04**	**3.87×10^{-4}**	**0.02**	**0.77**	**0.26**	**0.004**	**0.39**	**0.24**	**0.10**	**0.56**	**0.18**	**0.002**
6:151240811	rs7742959	intronic	*MTHFD1L*	G	0.02	0.14	0.04	4.13×10^{-4}	0.02	0.77	0.26	0.004	0.40	0.24	0.09	0.56	0.18	0.002
6:151240815	rs7742966	intronic	*MTHFD1L*	G	0.02	0.14	0.04	4.13×10^{-4}	0.02	0.77	0.26	0.004	0.40	0.24	0.09	0.56	0.18	0.002
6:151241076	rs9371203	intronic	*MTHFD1L*	G	0.02	0.14	0.04	4.13×10^{-4}	0.02	0.77	0.26	0.004	0.40	0.24	0.09	0.56	0.18	0.002
6:151241666	rs12189606	intronic	*MTHFD1L*	G	0.02	0.14	0.04	4.13×10^{-4}	0.02	0.73	0.26	0.006	0.38	0.24	0.11	0.53	0.18	0.003
7:24354300	rs4307239	intergenic	*NPY;MPP6*	G	0.71	-0.05	0.01	4.18×10^{-5}	0.70	-0.19	0.08	0.02	0.00	0.07	0.96	-0.09	0.05	0.12
8:75373309	rs10100113	intronic	*GDAP1*	G	0.55	-0.05	0.01	5.61×10^{-5}	0.55	-0.20	0.08	0.009	0.03	0.07	0.68	-0.08	0.05	0.12
**9:103824645**	**rs139371995**	**intronic**	***PLPPR1***	**C**	**0.03**	**0.15**	**0.04**	**3.47×10^{-5}**	**0.03**	**0.45**	**0.23**	**0.048**	**0.61**	**0.21**	**0.004**	**0.55**	**0.16**	**4.91×10^{-4}**
9:138631654	rs755722	intronic	*KCNT1*	T	0.37	-0.05	0.01	9.30×10^{-5}	0.35	-0.17	0.08	0.04	0.11	0.07	0.12	-0.02	0.05	0.76
9:138633407	rs11103153	intronic	*KCNT1*	T	0.38	-0.05	0.01	1.38×10^{-4}	0.35	-0.16	0.08	0.05	0.12	0.07	0.11	-0.02	0.05	0.83
10:13124513	rs11258178	intronic	*CCDC3*	A	0.58	0.05	0.01	1.12×10^{-5}	0.54	0.15	0.07	0.05	-0.10	0.07	0.13	0.01	0.05	0.80
**10:90980978**	**rs7910150**	**intronic**	***LIPA***	**A**	**0.83**	**-0.07**	**0.02**	**3.90×10^{-5}**	**0.80**	**-0.37**	**0.10**	**1.82×10^{-4}**	**0.06**	**0.09**	**0.54**	**-0.14**	**0.07**	**0.03**
11:86167136	rs2125362	intronic	*ME3*	A	0.77	0.07	0.01	3.66×10^{-7}	0.76	0.18	0.09	0.05	0.11	0.08	0.18	0.14	0.06	0.02
**11:86167202**	**rs2125363**	**intronic**	***ME3***	**G**	**0.77**	**0.07**	**0.01**	**3.65×10^{-7}**	**0.76**	**0.18**	**0.09**	**0.045**	**0.11**	**0.08**	**0.18**	**0.14**	**0.06**	**0.02**
12:102911650	rs35748	intergenic	*IGF1;LINC00485*	C	0.96	0.11	0.03	1.42×10^{-4}	0.95	0.35	0.17	0.04	0.01	0.15	0.94	0.16	0.12	0.16
13:90633423	rs9588824	intergenic	*LINC00353;LINC00559*	G	0.14	-0.07	0.02	7.15×10^{-5}	0.16	-0.30	0.11	0.005	0.04	0.10	0.65	-0.11	0.07	0.11
14:47222188	rs8018961	intergenic	*RPL10L;MDGA2*	T	0.86	0.07	0.02	9.17×10^{-5}	0.84	0.22	0.10	0.04	-0.09	0.10	0.34	0.05	0.07	0.51
14:47222965	rs12431742	intergenic	*RPL10L;MDGA2*	C	0.86	0.07	0.02	8.92×10^{-5}	0.84	0.22	0.10	0.04	-0.10	0.10	0.29	0.04	0.07	0.55
**14:72872021**	**rs10147214**	**intronic**	***RGS6***	**T**	**0.01**	**-0.33**	**0.08**	**6.42×10^{-5}**	**0.02**	**-0.67**	**0.32**	**0.04**	**-0.30**	**0.30**	**0.32**	**-0.46**	**0.22**	**0.04**
14:72872708	rs10148175	intronic	*RGS6*	C	0.01	-0.33	0.08	6.40×10^{-5}	0.02	-0.65	0.33	0.05	-0.29	0.30	0.34	-0.45	0.22	0.04
14:92795912	rs4904871	intronic	*SLC24A4*	G	0.51	-0.04	0.01	3.31×10^{-4}	0.55	-0.16	0.08	0.04	0.02	0.07	0.74	-0.06	0.05	0.25

© 2024 XU HM et al. *JAMA Network Open.*

Chr:Position[a]	rsID	ANNOVAR annotation	Closest gene(s)[b]	Effect allele	SIT[c]				CKB[c]									
									EAF	set-1			set-2			Total		
					EAF	Beta	SE	*P*		Beta	SE	*P*	Beta	SE	*P*	Beta	SE	*P*
16:68428740	rs9972857	intronic	*SMPD3*	T	0.07	-0.10	0.02	1.22×10^{-5}	0.08	-0.29	0.14	0.04	-0.09	0.13	0.49	-0.18	0.09	0.06
**16:69201758**	**rs1110549**	**intronic**	***UTP4***	**T**	**0.01**	**-0.36**	**0.08**	$\mathbf{3.25\times10^{-6}}$	**0.02**	**-1.10**	**0.45**	**0.01**	**-0.45**	**0.41**	**0.27**	**-0.74**	**0.30**	**0.02**
17:27711791	rs147778186	intergenic	*NUFIP2;TAOK1*	T	0.05	0.19	0.05	2.93×10^{-4}	0.05	0.38	0.19	0.05	0.01	0.17	0.97	0.17	0.13	0.18
19:24494914	rs7339681	intergenic	*HAVCR1P1;NONE*	A	0.05	-0.12	0.03	2.38×10^{-4}	0.05	-0.37	0.18	0.04	-0.21	0.17	0.21	-0.27	0.12	0.03
22:29334600	rs132556	intronic	*ZNRF3*	T	0.94	0.10	0.03	5.17×10^{-5}	0.95	0.35	0.16	0.03	0.08	0.15	0.57	0.20	0.11	0.06
22:29358219	rs5762916	intronic	*ZNRF3*	C	0.95	0.11	0.03	5.20×10^{-5}	0.95	0.34	0.16	0.04	0.11	0.15	0.47	0.21	0.11	0.06
**22:39405511**	**rs5995654**	**intergenic**	***APOBEC3B-AS1;APOBEC3C***	**A**	**0.35**	**-0.06**	**0.01**	$\mathbf{1.17\times10^{-5}}$	**0.32**	**-0.19**	**0.08**	**0.02**	**-0.09**	**0.07**	**0.21**	**-0.14**	**0.05**	**0.009**
22:39406322	rs7289061	intergenic	*APOBEC3B-AS1;APOBEC3C*	A	0.32	-0.05	0.01	4.22×10^{-5}	0.30	-0.18	0.08	0.03	-0.13	0.07	0.073	-0.16	0.06	0.004

[a] Reference as Genome Reference Consortium human genome (GRCh 37).
[b] Closest genes were annotated by ANNOVAR.
[c] A longitudinal genome-wide association analysis was performed in the SIT cohort using MAGEE, with gastric histological diagnoses (global severity score) at multiple time points as the study outcome. Then, association tests for individual genetic variants were performed based on the CKB set-1 using SAIGE, with the risk of gastric cancer as the study outcome. Analyses were conducted adjusting for age, sex, *H.pylori* infection status (for SIT only), regions (for CKB only) and principal components. SNPs with a *P*-value $<5\times10^{-4}$ in the longitudinal analysis and $P<0.05$ in the CKB set-1 are presented here. Lead SNPs at each genomic locus are bolded.
Abbreviations: Chr, chromosome; CKB, China Kadoorie Biobank; EAF, effect allele frequency; *H. pylori*, *Helicobacter pylori*; SIT, Shandong Intervention Trial; SNP, single nucleotide polymorphism.

© 2024 XU HM et al. *JAMA Network Open.*

eTable 5. Analysis for previously reported genome-wide significant SNPs in the GWAS-Catalog[a]

Chr	Position	rsID	Effect allele	EAF	Previously reported risk of GC		Risk for progression of gastric lesions in the SIT[c]		Risk for GC in the CKB[d]			
									set-1		set-2	
					OR	*P*[b]	Beta	*P*	Beta	*P*	Beta	*P*
1	155216951	rs1057941	A	NR	1.33	2×10^{-33}	-0.01	0.78	**0.31**	**8.40×10^{-4}**	**0.22**	**0.005**
1	156119243	rs138554234	A	0.05	0.64	4×10^{-15}	0.04	0.50	-0.26	0.11	-0.16	0.08
1	155192276	rs4072037	T	0.84	1.35	6×10^{-17}	0	0.85	**0.32**	**7.81×10^{-4}**	**0.18**	**0.03**
1	155171470	rs4971092	T	NR	0.68	1×10^{-8}	-0.07	0.46	-0.03	0.94	0.02	0.73
1	155199139	rs7366775	A	0.80	1.41	1×10^{-34}	0	0.93	**0.32**	**5.73×10^{-4}**	**0.22**	**0.03**
1	155208991	rs760077	T	0.85	1.39	2×10^{-40}	0	0.99	**0.32**	**0.001**	**0.35**	**0.001**
1	155515236	rs80142782	T	0.93	1.61	2×10^{-19}	0.01	0.87	**0.27**	**0.04**	**0.33**	**0.009**
2	234557214	rs1108143	G	0.11	1.23	3×10^{-7}	-0.04	0.10	-0.04	0.74	0.02	0.67
2	43131988	rs12471190	T	0.75	0.90	4×10^{-6}	-0.02	0.27	0.01	0.87	-0.07	0.12
3	10265247	rs2544001	T	0.98	0.74	5×10^{-6}	0.04	0.47	0.37	0.11	0.08	0.68
3	94389819	rs7624041	G	0.08	1.21	5×10^{-9}	-0.02	0.44	0.04	0.77	0.09	0.48
3	114643917	rs9841504	C	NR	1.32	2×10^{-9}	0	0.83	0.08	0.47	-0.06	0.52
4	124530209	rs10029005	A	0.33	1.14	3×10^{-11}	0.02	0.33	0.08	0.34	-0.03	0.66
4	124535968	rs11937064	A	0.67	0.87	9×10^{-10}	-0.01	0.39	-0.07	0.35	0.03	0.71
4	184323165	rs793885	T	0.41	0.90	2×10^{-6}	0.01	0.53	0.05	0.49	0.05	0.48
5	40790449	rs10074991	A	0.51	0.78	3×10^{-30}	-0.03	0.07	**-0.23**	**0.002**	**-0.15**	**0.03**
5	40623536	rs114080964	T	0.14	1.18	1×10^{-7}	-0.01	0.64	0.15	0.23	0.20	0.08
5	40791782	rs13361707	C	NR	1.41	8×10^{-29}	0.02	0.10	**0.23**	**0.002**	**0.15**	**0.03**
5	40960176	rs2675982	T	0.09	1.18	5×10^{-6}	0.01	0.72	**0.28**	**0.045**	-0.20	0.11
5	1295234	rs2853669	A	0.63	1.12	3×10^{-6}	0.01	0.72	**0.16**	**0.047**	0.07	0.33

© 2024 XU HM et al. *JAMA Network Open.*

Chr	Position	rsID	Effect allele	EAF	Previously reported risk of GC		Risk for progression of gastric lesions in the SIT[c]		Risk for GC in the CKB[d]			
									set-1		set-2	
					OR	P^b	Beta	*P*	Beta	*P*	Beta	*P*
5	40755466	rs3805495	T	0.58	0.85	9×10^{-20}	-0.03	0.09	**-0.23**	**0.002**	**-0.14**	**0.04**
**5**	**40726036**	**rs6897169**	**C**	**0.51**	**1.25**	$\mathbf{5\times10^{-12}}$	**0.03**	**0.03**	**0.21**	**0.007**	**0.15**	**0.03**
5	89607147	rs7712641	C	0.54	1.19	1×10^{-11}	-0.01	0.35	-0.09	0.26	-0.01	0.86
**6**	**28172515**	**rs16893741**	**T**	**0.85**	**0.87**	$\mathbf{2\times10^{-6}}$	**-0.05**	**0.03**	-0.06	0.52	0.02	0.42
**6**	**30610558**	**rs2267637**	**T**	**0.31**	**1.11**	$\mathbf{3\times10^{-6}}$	**0.04**	**0.009**	0.09	0.26	0.03	0.54
6	41037763	rs2294693	C	0.24	1.18	3×10^{-8}	-0.02	0.34	**0.28**	**0.001**	0.06	0.78
6	40568389	rs2494938	A	0.23	1.15	1×10^{-12}	-0.01	0.51	0.08	0.38	-0.03	0.25
6	28302807	rs2799081	T	0.69	1.11	4×10^{-6}	0.01	0.52	0.08	0.32	0.04	0.82
6	29475198	rs77454196	A	0.05	0.78	3×10^{-6}	-0.01	0.84	-0.03	0.89	0.22	0.02
6	41016523	rs9381024	T	0.23	1.14	1×10^{-7}	-0.02	0.30	**0.29**	**0.001**	-0.05	0.66
**6**	**27342754**	**rs9461366**	**A**	**0.15**	**1.15**	$\mathbf{2\times10^{-6}}$	**0.04**	**0.04**	0.04	0.71	0.10	0.10
7	17982637	rs11560253	A	0.75	1.12	4×10^{-6}	-0.02	0.31	0.01	0.92	0.02	0.82
7	21544470	rs2285947	A	0.27	1.17	1×10^{-16}	-0.02	0.41	-0.09	0.29	0.00	0.98
8	36806000	rs11775036	T	0.20	0.88	7×10^{-6}	-0.03	0.13	-0.14	0.15	0.01	0.92
8	142680513	rs2294008	T	NR	1.31	1×10^{-44}	-0.01	0.65	0.14	0.09	0.10	0.19
8	142695299	rs2585177	A	0.84	0.80	2×10^{-13}	-0.02	0.40	-0.18	0.11	-0.20	0.06
8	142682204	rs2976394	C	0.52	0.70	2×10^{-13}	0	0.80	-0.15	0.07	-0.11	0.16
8	142674302	rs2978977	A	0.49	1.29	3×10^{-43}	-0.01	0.67	0.23	0.02	0.16	0.09
9	6043593	rs343471	T	0.83	1.15	3×10^{-16}	-0.03	0.11	**-0.23**	**0.02**	0.06	0.54
9	133251249	rs7849280	G	NR	1.15	3×10^{-13}	0	0.98	**0.29**	**0.003**	0.08	0.38
10	94308190	rs10509670	A	0.79	0.78	2×10^{-22}	0.02	0.38	**-0.21**	**0.02**	-0.13	0.11
10	94309297	rs10509671	C	0.15	1.34	3×10^{-12}	0.02	0.23	0.17	0.10	0.10	0.25

© 2024 XU HM et al. *JAMA Network Open.*

Chr	Position	rsID	Effect allele	EAF	Previously reported risk of GC		Risk for progression of gastric lesions in the SIT[c]		Risk for GC in the CKB[d]			
									set-1		set-2	
					OR	*P*[b]	Beta	*P*	Beta	*P*	Beta	*P*
10	94310618	rs3781264	C	0.15	1.36	4×10^{-9}	0.02	0.24	0.17	0.10	0.10	0.29
11	133242868	rs4578395	C	0.09	1.19	2×10^{-6}	-0.01	0.70	-0.18	0.17	-0.22	0.07
12	58812181	rs11172733	T	0.84	0.87	1×10^{-6}	0.02	0.32	0.01	0.96	-0.14	0.13
14	36008748	rs11851309	T	0.38	0.89	3×10^{-7}	0	0.91	0.07	0.40	-0.04	0.61
**15**	**40502999**	**rs999197**	**T**	**0.11**	**1.18**	$\mathbf{1\times10^{-6}}$	**0.06**	**0.049**	0.05	0.70	0.07	0.51
16	11949655	rs3850997	T	0.30	1.15	2×10^{-9}	0	0.91	-0.01	0.87	-0.07	0.29
20	31411284	rs2376549	C	NR	1.11	8×10^{-10}	-0.01	0.63	0.04	0.63	-0.11	0.16
20	10098748	rs6039695	A	0.34	0.89	1×10^{-6}	0	0.93	0.06	0.45	-0.06	0.40

[a] Up to December 31, 2022, a total of 67 variants were associated with GC risk for Asian populations based on GWAS-catalog, 50 of which were genotyped/imputed and passed quality control in the Shandong Intervention Trial cohort.
[b] *P*-values were obtained from GWAS-catalog which does not provide more digits to the right of the decimal point.
[c]A longitudinal genome-wide association analysis was performed in the SIT cohort using MAGEE, with gastric histological diagnoses (global severity score) at multiple time points as the study outcome. Analyses were conducted adjusting for age, sex, *H.pylori* infection status, and principal components. SNPs with $P<0.05$ and consistent directions of association are bolded.
[d] Analyses were conducted to examine GC risk associated with each genetic variant in the CKB set-1 and set-2 using SAIGE. Analyses were conducted adjusting for age, sex, regions and principal components. SNPs with $P<0.05$ and consistent directions of association are bolded.
Abbreviations: Chr, chromosome; CKB, China Kadoorie Biobank; EAF, effect allele frequency; GC, gastric cancer; GWAS, genome-wide association study; NR, not reported; OR, odds ratio; SIT, Shandong Intervention Trial; SNP, single nucleotide polymorphism.

© 2024 XU HM et al. *JAMA Network Open.*

eTable 6. Sensitivity analyses of 12 gastric cancer-related SNPs based on the Shandong Intervention Trial.

Locus[a]	rsID	Effect allele	EAF	Original result			Sensitivity analysis only having four-times diagnoses during follow-up [b]			Sensitivity analysis by additionally adjusting for three chemopreventions[c]		
				Beta	SE	*P*	Beta	SE	*P*	Beta	SE	*P*
1p31.1	rs12070840	T	0.06	0.09	0.02	2.40×10^{-4}	0.10	0.04	0.009	0.10	0.02	1.39×10^{-5}
1p21.1	rs78078728	T	0.01	-0.26	0.06	3.91×10^{-5}	-0.19	0.10	0.06	-0.2	0.06	1.37×10^{-5}
4q12	rs41481345	G	0.05	0.10	0.03	5.82×10^{-5}	0.09	0.04	0.03	0.10	0.02	4.32×10^{-5}
4q28.2	rs2391536	G	0.95	-0.08	0.03	3.91×10^{-4}	-0.11	0.04	0.01	-0.07	0.03	3.21×10^{-3}
5q35.1	rs6879467	G	0.53	-0.06	0.01	1.71×10^{-6}	-0.07	0.02	1.46×10^{-4}	-0.06	0.01	7.32×10^{-7}
6q25.1	rs9478852	G	0.02	0.14	0.04	3.87×10^{-4}	0.10	0.06	0.11	0.14	0.04	8.88×10^{-5}
9q31.1	rs139371995	C	0.03	0.15	0.04	3.47×10^{-5}	0.17	0.06	0.002	0.14	0.05	6.22×10^{-6}
10q23.31	rs7910150	A	0.83	-0.07	0.02	3.90×10^{-5}	0.06	0.03	0.02	-0.07	0.01	2.08×10^{-6}
11q14.2	rs2125363	G	0.77	0.07	0.01	3.65×10^{-7}	-0.10	0.02	8.02×10^{-6}	0.06	0.01	3.14×10^{-7}
14q24.2	rs10147214	T	0.01	-0.33	0.08	6.42×10^{-5}	-0.28	0.13	0.03	-0.33	0.07	1.02×10^{-5}
16q22.1	rs1110549	T	0.01	-0.36	0.08	3.25×10^{-6}	-0.44	0.12	2.09×10^{-4}	-0.36	0.07	1.94×10^{-7}
22q13.1	rs5995654	A	0.35	-0.06	0.01	1.17×10^{-5}	-0.07	0.02	2.32×10^{-4}	-0.06	0.01	6.26×10^{-7}

[a] Annotated by ANNOVAR cytoband database.
[b] While the primary analysis utilized the histopathological diagnoses of all five times, a sensitivity analysis was conducted by only having four-times diagnoses during follow-up (1994, 1999, 2003 and 2022) as the outcome variable in the longitudinal matrix of generalized linear mixed models and adjusting for baseline diagnosis (1989), along with age, sex, *H. pylori* infection status, and principal components as covariates.
[c] Sensitivity analysis was also conducted by additionally adjusting for three chemopreventions, including *H. pylori* treatment (without attending the intervention trial in 1995, *H. pylori* negative, *H. pylori* positive receiving active treatment, or *H. pylori* positive receiving placebo), vitamin supplementation (without attending the intervention trial in 1995, receiving active treatment, or receiving placebo) and garlic supplementation (without attending the intervention trial in 1995, receiving active treatment, or receiving placebo), along with age, sex, and principal components as covariates.
Abbreviations: EAF, effect allele frequency; *H. pylori*, *Helicobacter pylori*; SNP, single nucleotide polymorphism.

© 2024 XU HM et al. *JAMA Network Open.*

eTable 7. Sensitivity analysis of polygenic risk scores associated with the risk of gastric cancer in the SIT, CKB, and external case-control validation set

	SIT				CKB										Case-control validation set		
					set-1			set-2			Total						
	No. of cases (person-years)	Age-adjusted IR/100,000 PYs	HR (95% CI)[a]	*P*	No. of cases (person-years)	HR (95% CI)[a]	*P*	No. of cases (person-years)	HR (95% CI)[a]	*P*	No. of cases (person-years)	Age-adjusted IR/100,000 PYs	HR (95% CI)[a]	*P*	No. of cases /controls	OR (95% CI)	*P*
Decile 1	7 (8,758)	89	Ref		12 (61,222)	Ref		35 (60,293)	Ref		47 (121,515)	39	Ref		63/77	Ref	
Decile 2	7 (7,720)	82	1.13 (0.40-3.20)	0.82	25 (55,139)	2.26 (1.13-4.50)	0.02	47 (55,597)	1.45 (0.93-2.25)	0.10	71 (110,417)	65	1.65 (1.14-2.39)	0.008	75/74	1.43 (0.87-2.35)	0.16
Decile 3	12 (7,785)	159	1.83 (0.72-4.64)	0.20	26 (66,064)	1.96 (0.99-3.89)	0.05	56 (66,820)	1.47 (0.96-2.24)	0.07	83 (133,203)	62	1.59 (1.11-2.28)	0.01	80/86	1.18 (0.73-1.91)	0.51
Decile 4	10 (8,280)	134	1.53 (0.58-4.02)	0.39	33 (48,322)	3.28 (1.69-6.36)	<0.001	32 (49,529)	1.12 (0.69-1.81)	0.64	65 (97,151)	67	1.67 (1.15-2.44)	0.007	49/55	1.24 (0.72-2.13)	0.44
Decile 5	7 (7,667)	104	1.17 (0.41-3.35)	0.77	41 (60,178)	3.37 (1.77-6.41)	<0.001	44 (61,320)	1.24 (0.80-1.93)	0.35	85 (122,199)	70	1.78 (1.25-2.55)	0.002	75/64	1.53 (0.92-2.54)	0.10
Decile 6	15 (7,883)	242	2.23 (0.93-5.66)	0.07	30 (55,046)	2.69 (1.37-5.27)	0.004	41 (54,194)	1.38 (0.88-2.15)	0.16	72 (109,729)	65	1.71 (1.19-2.47)	0.004	71/76	1.32 (0.80-2.17)	0.28
Decile 7	12 (8,044)	186	1.78 (0.70-4.53)	0.22	43 (57,670)	3.66 (193-6.95)	<0.001	44 (57,874)	1.24 (0.79-1.95)	0.34	84 (114,689)	73	1.86 (1.30-2.66)	<0.001	69/62	1.46 (0.88-2.44)	0.15
Decile 8	15 (8,081)	165	2.42 (0.99-5.92)	0.05	48 (60,748)	3.98 (2.12-7.48)	<0.001	51 (65,090)	1.38 (0.90-2.12)	0.14	101 (126,203)	80	2.04 (1.45-2.89)	<0.001	64/76	1.15 (0.69-1.90)	0.60
Decile 9	27 (7,778)	372	4.11 (1.79-9.46)	<0.001	56 (54,105)	5.04 (2.70-9.41)	<0.001	49 (51,438)	1.67 (1.08-2.58)	0.02	105 (105,543)	98	2.53 (1.79-3.57)	<0.001	75/64	1.86 (1.12-3.10)	0.02
Decile 10	35 (7,612)	486	5.58 (2.46-12.59)	<0.001	61 (56,963)	5.25 (2.83-9.76)	<0.001	51 (57,578)	1.51 (0.98-2.58)	0.06	112 (114,541)	98	2.46 (1.75-3.46)	<0.001	81/59	1.81 (1.10-3.01)	0.02
Per one SD score	147 (79,608)	199	1.69 (1.47-1.94)	<0.001	375 (575,456)	1.45 (1.33-1.59)	<0.001	450 (579,734)	1.13 (1.02-1.22)	0.02	825 (1,155,190)	71	1.26 (1.18-1.34)	<0.001	702/692	1.14 (1.02-1.28)	0.02

© 2024 XU HM et al. *JAMA Network Open.*

[a] Sensitivity analyses were conducted by adjusting for age, sex, *H. pylori* infection status (for SIT only), regions (for CKB only), without adjusting for principal components based on the Fine-Gray model (SIT and CKB cohort) and logistic regression model (external case-control validation set) as appropriate for the design of each study.
Abbreviations: CI, confidence interval; CKB, China Kadoorie Biobank; HR, hazard ratio; IR, incidence ratio; OR, odds ratio; SD, standard deviation; SIT, Shandong Intervention Trial.

© 2024 XU HM et al. *JAMA Network Open.*

eTable 8. Sensitivity analyses of *H.pylori* treatment and nutrition supplementation associated with incident gastric cancer by different genetic-risks based on the Shandong Intervention Trial

Group	No. of cases (person-years)		Primary analyses		Sensitivity analysis without adjusting for PCs[b]		Sensitivity analysis by excluding SNPs with MAF≤2% for PRS construction[c]			
			HR (95% CI)[a]	*P* for interaction	HR (95% CI)[a]	*P for* interaction	No.of cases (person-years)		HR (95% CI)[a]	*P for* interaction
	Placebo	Treatment					Placebo	Treatment		
H. pylori treatment										
Low genetic risk	36 (16,846)	28 (16,206)	0.81 (0.50-1.34)	0.03	0.81 (0.49-1.33)	0.03	36 (16,895)	25 (16,103)	0.74 (0.44-1.24)	0.02
High genetic risk	35 (5,738)	15 (5,300)	0.45 (0.24-0.82)		0.45 (0.25-0.83)		35 (5,689)	18 (5,403)	0.50 (0.28-0.89)	
Vitamin supplementation										
Low genetic risk	42 (23,862)	33 (23,245)	0.84 (0.53-1.33)	0.93	0.83 (0.52-1.31)	0.90	40 (23,748)	32 (23,106)	0.86 (0.54-1.37)	0.95
High genetic risk	34 (7,461)	30 (7,393)	0.85 (0.51-1.42)		0.86 (0.53-1.41)		36 (7,575)	31 (7,533)	0.83 (0.50-1.36)	
Garlic supplementation										
Low genetic risk	38 (23,491)	37 (23,617)	1.02 (0.65-1.62)	0.41	0.97 (0.62-1.53)	0.41	38 (23,429)	34 (23,425)	0.92 (0.58-1.47)	0.73
High genetic risk	38 (7,746)	26 (7,107)	0.72 (0.43-1.22)		0.73 (0.44-1.20)		38 (7,809)	29 (7,299)	0.79 (0.47-1.31)	

[a]The HRs (95% CIs) were computed using Fine-Gray regression models adjusting for age, sex, *H.pylori* infection status and principal components. *P* values for interaction were obtained by incorporating a multiplicative term of the examined intervention and genetic risk variables into the Fine-Gray models.
[b]Analyses were conducted by adjusting for age, sex, and *H. pylori* infection, without adjusting for PCs based on the Shandong Intervention Trial participants.
[c] Analyses were conducted after exclusion of three SNPs with MAF≤2% in the SIT participants (rs78078728, rs10147214, and rs1110549).
Abbreviations: CI, confidence interval; HR, hazard ratio; MAF, minor allele frequency, PCs, principal components; PRS, polygenic risk score.

© 2024 XU HM et al. *JAMA Network Open.*

eTable 9. Differentially expressed gene(s) mapped within 1000Kb of each genomic locus in stomach tissues

Lead SNP	Genomic locus[a]	No. of candidate SNPs[b]	Gene[c]	Genes expressed in gastric tissues[d]		Differentially expressed gene[e]		
				Average TPM in normal stomach tissues	Genes expressed in gastric tissues	Log_2Fold Change	*P*	Regulated
rs12070840	1:82219539-82330750	62	*LPHN2*	N.A.	N.A.			
rs78078728	1:103288854-103775966	8	*RNPC3*	6.55	1	-1.51	$1.15×10^{-80}$	normal
			AMY2A	N.A.	N.A.			
			AMY1A	N.A.	N.A.			
			AMY1B	N.A.	N.A.			
			AMY1C	N.A.	N.A.			
			COL11A1	0.008	0			
			OLFM3	0.006	0			
			AMY2B	1.56	1	-2.65	$6.03×10^{-81}$	normal
rs41481345	4:55941418-55944618	5	*KIT*	1.60	1	-2.45	$1.04×10^{-87}$	normal
			EXOC1	6.58	1	-0.06	0.15	normal
			KDR	2.71	1	-0.07	0.34	normal
			PDGFRA	11.19	1	-1.07	$1.75×10^{-25}$	normal
			TMEM165	29.85	1	0.21	$1.98×10^{-6}$	normal
			CEP135	0.82	0			
			PDCL2	N.A.	N.A.			
			FIP1L1	3.79	1	0	0.88	normal
			SRD5A3	6.84	1	1.48	$2.60×10^{-51}$	normal
			CLOCK	11.47	1	0.34	$1.57×10^{-12}$	normal
			NMU	4.60	1	3.67	$6.53×10^{-44}$	normal
			GSX2	0	0			
rs2391536	4:130596796-130720635	19	*JADE1*	4.73	1	-0.70	$8.33×10^{-27}$	normal
			SCLT1	3.92	1	0.50	$5.59×10^{-25}$	normal
			C4orf33	2.69	1	0.22	0.002	normal
rs6879467	5:169088449-169110947	15	*LCP2*	3.30	1	0.97	$1.83×10^{-25}$	normal
			DOCK2	4.02	1	-0.23	0.04	normal
			KCNMB1	0.65	0			
			SPDL1	2.37	1	1.27	$3.58×10^{-65}$	normal
			KCNIP1	0.16	0			
			FAM196B	N.A.	N.A.			
			FOXI1	0.01	0			
			SLIT3	1.28	1	-0.73	$4.26×10^{-8}$	normal
			C5orf58	0.07	0			
rs9478852	6:151232825-151247079	41	*RMND1*	6.24	1	0.42	$3.93×10^{-16}$	normal
			PPP1R14C	0.41	0			
			PLEKHG1	5.60	1	1.30	$1.24×10^{-59}$	normal
			IYD	0.94	0			

© 2024 XU HM et al. *JAMA Network Open.*

Lead SNP	Genomic locus[a]	No. of candidate SNPs[b]	Gene[c]	Genes expressed in gastric tissues[d]		Differentially expressed gene[e]		
				Average TPM in normal stomach tissues	Genes expressed in gastric tissues	Log_2Fold Change	*P*	Regulated
rs9478852	6:151232825-151247079	41	*ULBP3*	1.06	1	2.67	1.30×10^{-57}	normal
			C6orf211	N.A.	N.A.			
			ULBP1	0.01	0			
			RAET1G	0.15	0			
			ZBTB2	4.50	1	0.65	1.29×10^{-49}	normal
			CCDC170	0.48	0			
			RAET1L	0.00	0			
			MTHFD1L	4.23	1	1.80	5.42×10^{-89}	normal
			AKAP12	1.05	1	-1.17	1.55×10^{-16}	normal
			ESR1	0.88	0			
			ULBP2	0.11	0			
rs139371995	9:103556287-103839276	6	*MSANTD3*	2.35	1	0.53	2.36×10^{-25}	normal
			ALDOB	15.87	1	0.04	0.90	normal
			GRIN3A	0.04	0			
			RNF20	4.16	1	0.41	3.68×10^{-25}	normal
			LPPR1	N.A.	N.A.			
			TMEM246	N.A.	N.A.			
			TEX10	2.76	1	0.22	3.86×10^{-7}	normal
			MRPL50	1.42	1	0.58	2.80×10^{-27}	normal
			ZNF189	4.51	1	-0.05	0.36	normal
			MSANTD3-TMEFF1	0.24	0			
			ERP44	7.85	1	0.51	8.16×10^{-27}	normal
			BAAT	0.01	0			
			INVS	7.25	1	0.25	3.40×10^{-8}	normal
			STX17	3.84	1	0.06	0.14	normal
			TMEFF1	0.10	0			
			NR4A3	0.69	0			
			PPP3R2	0	0			
rs7910150	10:90905561-90986063	110	*CH25H*	0.37	0			
			KIF20B	0.96	0			
			LIPF	33.61	1	-9.07	2.28×10^{-64}	down
			IFIT1	1.35	1	1.09	1.95×10^{-14}	normal
			PANK1	2.76	1	0.79	5.42×10^{-18}	normal
			FAS	0.99	0			
			LIPJ	0.01	0			
			SLC16A12	0.09	0			
			RNLS	0.31	0			
			LIPM	0.04	0			
			LIPK	N.A.	N.A.			
			IFIT5	1.88	1	0.67	2.44×10^{-23}	normal
			LIPN	0	0			
			ACTA2	41.51	1	-1.48	8.98×10^{-26}	normal

© 2024 XU HM et al. *JAMA Network Open.*

Lead SNP	Genomic locus[a]	No. of candidate SNPs[b]	Gene[c]	Genes expressed in gastric tissues[d]		Differentially expressed gene[e]		
				Average TPM in normal stomach tissues	Genes expressed in gastric tissues	Log_2Fold Change	*P*	Regulated
rs7910150	10:90905561-90986063	110	*IFIT3*	4.24	1	1.44	3.31×10^{-34}	normal
			LIPA	6.90	1	1.24	1.37×10^{-49}	normal
			STAMBPL1	5.82	1	0.68	2.61×10^{-17}	normal
			ANKRD22	3.80	1	0.26	0.22	normal
			IFIT1B	0	0			
			IFIT2	1.32	1	1.43	3.14×10^{-32}	normal
rs2125363	11:86101234-86199583	55	*FZD4*	2.04	1	-1.26	9.20×10^{-55}	normal
			TMEM135	1.82	1	0.73	4.52×10^{-36}	normal
			PRSS23	8.49	1	-0.55	6.08×10^{-10}	normal
			DLG2	0.73	0			
			CREBZF	11.91	1	-1.23	8.61×10^{-58}	normal
			AP000974.1	N.A.	N.A.			
			C11orf73	N.A.	N.A.			
			CCDC89	1.86	1	-0.96	5.62×10^{-22}	normal
			TMEM126A	2.61	1	0	0.95	normal
			PICALM	18.80	1	0.36	1.84×10^{-15}	normal
			EED	2.82	1	0.05	0.22	normal
			ME3	9.08	1	-1.75	6.52×10^{-95}	normal
			CCDC81	0.28	0			
			SYTL2	76.12	1	-0.25	0.02	normal
			CCDC83	0.00	0			
			TMEM126B	4.25	1	-0.02	0.67	normal
rs10147214	14:72859949-72876360	4	*ZFYVE1*	5.59	1	-0.46	4.38×10^{-25}	normal
			DCAF4	3.04	1	-0.30	1.72×10^{-9}	normal
			AC005477.1	N.A.	N.A.			
			RGS6	0.64	0			
			PSEN1	19.59	1	0.59	4.80×10^{-44}	normal
			DPF3	6.08	1	-0.75	4.66×10^{-17}	normal
			PAPLN	3.41	1	-0.85	1.65×10^{-14}	normal
			RBM25	30.68	1	-0.39	3.83×10^{-11}	normal
			NUMB	34.82	1	-0.06	0.10	normal
			SIPA1L1	24.87	1	0.27	5.68×10^{-9}	normal
rs1110549	16:69201758-69201758	1	*ZFP90*	1.91	1	-0.08	0.07	normal
			SMPD3	74.33	1	-0.19	0.20	normal
			NOB1	14.28	1	0.38	3.10×10^{-13}	normal
			VPS4A	10.44	1	-0.61	8.89×10^{-43}	normal
			TANGO6	2.76	1	-0.01	0.78	normal
			TMED6	1.07	1	-2.73	1.83×10^{-40}	down
			PDF	2.94	1	1.15	1.06×10^{-43}	normal
			SLC7A6	3.22	1	-0.17	0.02	normal
			HAS3	2.81	1	-0.15	0.37	normal
			SNTB2	4.15	1	0.16	7.67×10^{-4}	normal
			SLC7A6OS	4.97	1	-0.41	1.64×10^{-22}	normal
rs1110549		1	*CDH1*	107.95	1	2.07	1.63×10^{-25}	normal

© 2024 XU HM et al. *JAMA Network Open.*

Lead SNP	Genomic locus[a]	No. of candidate SNPs[b]	Gene[c]	Genes expressed in gastric tissues[d]		Differentially expressed gene[e]		
				Average TPM in normal stomach tissues	Genes expressed in gastric tissues	Log_2Fold Change	*P*	Regulated
			WWP2	29.86	1	0	0.99	normal
			NIP7	3.44	1	1.06	2.07×10^{-68}	normal
			CDH3	3.14	1	3.67	4.07×10^{-70}	up
			CLEC18A	1.39	1	-1.35	1.59×10^{-18}	normal
			COG8	13.11	1	0.05	0.20	normal
			CIRH1A	N.A.	N.A.			
			RP11-343C2.11	N.A.	N.A.			
			RP11-343C2.12	N.A.	N.A.			
			RP11-343C2.9	N.A.	N.A.			
	16:69201758-69201758		*RP11-343C2.7*	N.A.	N.A.			
			TERF2	9.44	1	0.42	5.17×10^{-25}	normal
			PDPR	6.00	1	-0.47	1.97×10^{-13}	normal
			PRMT7	7.70	1	-0.32	1.31×10^{-13}	normal
			NFATC3	5.91	1	0.95	3.86×10^{-55}	normal
			PLA2G15	4.35	1	0.45	2.42×10^{-19}	normal
			NQO1	107.75	1	0.15	0.30	normal
			ESRP2	40.97	1	0.94	6.77×10^{-8}	normal
			NFAT5	11.24	1	0.36	1.47×10^{-8}	normal
			CYB5B	7.05	1	1.11	6.37×10^{-63}	normal
			CHTF8	12.40	1	-0.02	0.64	normal
			APOBEC3C	6.12	1	0.47	4.59×10^{-8}	normal
			CBX6	9.26	1	-1.00	6.80×10^{-22}	normal
			APOBEC3G	3.10	1	0.93	6.67×10^{-18}	normal
			APOBEC3D	4.12	1	0.43	6.87×10^{-6}	normal
			MAFF	7.50	1	-0.70	8.57×10^{-16}	normal
			APOBEC3F	2.51	1	1.03	4.91×10^{-33}	normal
			BAIAP2L2	47.52	1	2.83	2.19×10^{-56}	up
			APOBEC3H	0.21	0			
			GRAP2	1.29	1	-0.04	0.69	normal
			NPTXR	0.57	0			
rs5995654	22:39369272-39408841	54	*SYNGR1*	1.59	1	-2.21	6.82×10^{-73}	normal
			MGAT3	16.53	1	0.37	1.24×10^{-3}	normal
			MIEF1	8.99	1	0.35	4.49×10^{-18}	normal
			JOSD1	19.82	1	0.03	0.43	normal
			PICK1	16.26	1	-0.88	1.76×10^{-48}	normal
			DDX17	114.43	1	-1.11	2.56×10^{-53}	normal
			DMC1	0.30	0			
			APOBEC3B	2.11	1	2.68	3.73×10^{-42}	up
			SOX10	0.43	0			
			CBX7	10.24	1	-2.16	7.84×10^{-102}	normal
			AL031590.1	N.A.	N.A.			

© 2024 XU HM et al. *JAMA Network Open.*

Lead SNP	Genomic locus[a]	No. of candidate SNPs[b]	Gene[c]	Genes expressed in gastric tissues[d]		Differentially expressed gene[e]		
				Average TPM in normal stomach tissues	Genes expressed in gastric tissues	Log_2Fold Change	*P*	Regulated
			RPS19BP1	N.A.	N.A.			
rs5995654	22:39369272-39408841	54	*PLA2G6*	11.23	1	-1.25	$9.34×10^{-66}$	normal
			GTPBP1	32.60	1	-0.07	0.11	normal
			CSNK1E	32.24	1	-0.29	$1.44×10^{-9}$	normal
			PDGFB	1.58	1	0.45	$8.29×10^{-10}$	normal
			SLC16A8	1.78	1	-0.30	0.005	normal
			CACNA1I	0.07	0			
			KCNJ4	0	0			
			SUN2	39.10	1	-1.35	$4.61×10^{-80}$	normal
			CBY1	5.96	1	-0.75	$1.70×10^{-47}$	normal
			TAB1	16.90	1	-0.47	$9.56×10^{-30}$	normal
			DNAL4	6.82	1	-0.67	$6.90×10^{-33}$	normal
			FAM83F	4.99	1	0.21	0.22	normal
			FAM227A	0.42	0			
			APOBEC3A	0.68	0			
			POLR2F	4.75	1	0.07	0.17	normal
			TMEM184B	33.58	1	0.29	$1.15×10^{-10}$	normal
			ENTHD1	8.53	1	1.02	$3.47×10^{-15}$	normal
			RPS19BP1	36.87	1	-0.78	$1.11×10^{-50}$	normal
			TOMM22	8.37	1	0.05	0.27	normal
			KDELR3	19.54	1	2.10	$8.62×10^{-73}$	normal
			RPL3	490.95	1	-0.35	$1.11×10^{-8}$	normal
			ATF4	136.85	1	-1.38	$4.91×10^{-94}$	normal

[a]A genomic locus was defined as the LD block with a maximum distance of 250Kb from each independent significant SNP.
[b] The number of candidate SNPs in the genomic locus, including all SNPs that are in LD (r^2>0.4) with each independent significant SNP.
[c] Positional mapping of 188 genes was based on ANNOVAR annotations by specifying the maximum distance of 1000Kb between SNPs in genomic locus and genes.
[d] Of 188 genes, 166 genes were included in the RNA-Seq data for 88 non-gastric cancer tissues from Linqu county, Shandong province. Genes not included in the RNA-seq data are labeled as N.A.. Genes with transcript per million (TPM) >1 at more than 50% of samples were defined as genes expressed in gastric tissues, and are labeled as 1 here, otherwise are labeled as 0.
[e]123 genes expressed in gastric tissues were identified, all of which were available in the GTEx and TCGA-STAD data. Differentially expressed gene analyses were performed using limma, with Log_2FoldChange representing the difference in the logarithm base 2 transformed expression levels of a gene between gastric cancer tumor tissue (TCGA-STAD, n=413) and normal (GTEx, n=174) or adjacent non-cancer tissue (TCGA-STAD, n=36). Genes with log_2FoldChange>2.64 and $P<4.07×10^{-4}$ (Bonferroni correction, $4.07×10^{-4}$= 0.05/123 genes) were defined as upregulated, while genes with log_2FoldChange≤-2.64 and $P<4.07×10^{-4}$ were defined as downregulated DEGs in gastric cancer tumor vs non-tumor tissues. Genes with -2.64< log_2FoldChange <2.64 or *P* value beyond the significance threshold are marked as "normal" in the table.
Abbreviations: DEGs, differentially expressed genes; LD, linkage disequilibrium; N.A., not available; SNP, single nucleotide polymorphism.

© 2024 XU HM et al. *JAMA Network Open.*

eTable 10. KEGG network enrichment analysis for 123 genes expressed in gastric tissues with potential biological significance.

Pathway	ID	Input number	Background number	Enriched ratio (%)	*P*-Value	Corrected *P*-Value	Input
Rap1 signaling pathway	hsa04015	7	210	3.33	5.80×10^{-6}	3.60×10^{-4}	*PDGFB|PDGFRA|KIT|SIPA1L1|CDH1|LCP2| KDR*
Human immunodeficiency virus 1 infection	hsa05170	7	212	3.30	6.16×10^{-6}	3.66×10^{-4}	*NFATC3|APOBEC3D|APOBEC3G|APOBEC3F|APOBEC3C|APOBEC3B|TAB1*
Wnt signaling pathway	hsa04310	6	160	3.75	1.45×10^{-5}	7.60×10^{-4}	*NFATC3|FZD4|PSEN1|INVS|CSNK1E|CBY1*
MAPK signaling pathway	hsa04010	7	295	2.37	4.85×10^{-5}	2.20×10^{-4}	*PDGFB|NFATC3|TAB1|PDGFRA|KIT|ATF4|KDR*
Ras signaling pathway	hsa04014	5	232	2.16	0.001	0.02	*PLA2G6|PDGFB|PDGFRA|KDR|KIT*
Melanoma	hsa05218	3	72	4.17	0.002	0.03	*CDH1|PDGFB|PDGFRA*
EGFR tyrosine kinase inhibitor resistance	hsa01521	3	79	3.80	0.002	0.03	*PDGFB|PDGFRA|KDR*
Prostate cancer	hsa05215	3	97	3.09	0.004	0.04	*ATF4|PDGFB|PDGFRA*
T cell receptor signaling pathway	hsa04660	3	103	2.91	0.005	0.047	*LCP2|NFATC3|GRAP2*
Circadian rhythm	hsa04710	2	31	6.45	0.005	0.049	*CSNK1E|CLOCK*

ID: Pathway ID in the original database, Input number: The number of input genes within this functional term, Background number: The number of background genes within this functional term, Enriched Ratio (%)= (Input number / Background number) × 100, *P*: The calculated *P*, Corrected *P*: Multiple test correction was performed to calculate the false discovery rate *P* values. Pathways with corrected *P*<0.05 are shown.

eTable 11. The identification of differentially expressed genes between advanced and mild gastric lesions.

Genomic Locus	Genes[a]	IM/DYS vs.SG/CAG	
		Log2FoldChange[b]	*P*
1:103288854-103775966	*AMY2B*	0.07	0.70
4:55941418-55944618	*NMU*	-0.02	0.93
6:151232825-151247079	*ULBP3*	0.14	0.25
10:90905561-90986063	*LIPF*	-0.70	0.31
16:69201758-69201758	***CDH3***	**1.53**	**1.71×10^{-5}**
	TMED6	**-0.70**	**0.04**
22:39369272-39408841	***BAIAP2L2***	**0.94**	**2.17×10^{-6}**
	APOBEC3B	0.26	0.46

[a] 8 DEGs were identified based on TCGA/GTEx in the longitudinal genome-wide association analysis, all of which were available in the Linqu RNA-seq data.
[b] The analyses were performed using limma. Log_2FoldChange represented the difference in the logarithm base 2 transformed expression levels of a gene between advanced gastric lesion specimen (n=30) and mild gastric lesion specimen (n=54).
Abbreviations: CAG, chronic atrophic gastritis; DEGs, differentially expressed genes; DYS, dysplasia; IM, intestinal metaplasia; SG, superficial gastritis.

eTable 12. Cis-eQTL analysis based on analysis of Linqu stomach tissue-based datasets.

Locus	rsID[a]	Chr	Position	Gene Symbol	Tested allele	t-statistic[b]	*P*-value
22:39369272-39408841	rs36120190	22	39397847	*BAIAP2L2*	C	2.39	0.02
	rs9607601	22	39399438	*BAIAP2L2*	T	2.32	0.02
	rs61463331	22	39390231	*BAIAP2L2*	G	2.31	0.02
	rs6001364	22	39393332	*BAIAP2L2*	A	2.31	0.02
	rs12159666	22	39395144	*BAIAP2L2*	G	2.31	0.02
	rs9611069	22	39395612	*BAIAP2L2*	A	2.31	0.02
	rs6001367	22	39400527	*BAIAP2L2*	A	2.09	0.04
	rs6001368	22	39400832	*BAIAP2L2*	A	2.09	0.04
	rs6519161	22	39402450	*BAIAP2L2*	T	2.08	0.04
	rs5995653	22	39404249	*BAIAP2L2*	A	2.08	0.04
	rs6001370	22	39404635	*BAIAP2L2*	G	2.02	0.048

[a] Of 173 variants that were identified based on fine-mapping of regions around 8 differentially expressed genes, 11 were found to be cis-eQTLs. For space limitation, we only present cis-eQTLs with $P<0.05$.

[b] The R package "MatrixEQTL" was used for the analyses, with t-statistic as effect estimates and $P<0.05$ as significant. Non-gastric cancer subjects with genotype and RNA-seq data (n=65) were included for the analyses.

Abbreviations: Chr, chromosome; eQTL, expression quantitative trait loci.

© 2024 XU HM et al. *JAMA Network Open.*

eTable 13. Functional annotation for rs5995654 and its nearby variants[a]

Candidate variant	r^2	Pos	EA	RA	GWAS results[b]						Annovar Function	CADD PHRED[c]	RDB Score[d]	Prioritization score[e]	HaploReg Evidence					RDB evidence		
					SIT			CKB														
					MAF	Beta	*P*-value	MAF	Beta	*P*-value					Promoter Histone Marks	Enhancer histone marks	DNase	Proteins bound	Motifs changed	eQTLs	TF binding	DNase peak
rs2072866	0.62	39385809	C	G	N.A.	N.A.	N.A.	0.42	-0.09	0.13	intronic	1.19	5	**6**		BLD, MUS, THYM	THYM		Maf,Smad	F	T	T
rs760726	0.53	39388827	T	C	0.50	0.03	0.10	0.49	-0.09	0.07	ncRNA_intronic	3.77	5	**5**		6 tissues	IPSC		ERalpha-a,Pax-6,RAR	F	T	T
rs2010264	0.53	39388993	T	C	0.50	0.03	0.11	0.49	-0.09	0.07	ncRNA_exonic	1.00	5	**5**		6 tissues	7 tissues		SMC3,T3R	F	T	T
rs2019907	0.53	39389420	G	A	0.50	0.03	0.10	0.49	-0.09	0.06	ncRNA_intronic	4.14	**1f**	**7**	IPSC	8 tissues	4 tissues		Bcl6b,STAT	T	T	T
rs9607599	0.53	39389663	T	G	0.50	0.03	0.10	0.49	-0.09	0.07	ncRNA_intronic	5.63	**4**	**6**		6 tissues	4 tissues	TCF12	5 altered motifs	F	T	T
rs6001359	0.53	39389952	G	A	0.50	0.03	0.10	0.49	-0.09	0.07	ncRNA_intronic	5.11	6	**3**		IPSC, THYM, PANC			Pou2f2,Pou3f2	F	F	T
rs61463331	0.83	39390231	G	A	0.34	-0.06	7.76×10^{-4}	0.32	-0.13	0.02	ncRNA_intronic	6.72	5	**7**		IPSC, THYM, PANC	IPSC		4 altered motifs	F	T	T
rs6001360	0.53	39390661	G	A	0.50	0.03	0.11	0.48	-0.09	0.08	ncRNA_intronic	0.09	7	**1**						F	F	T
rs9611067	0.53	39390876	G	A	0.50	0.03	0.11	0.48	-0.09	0.08	ncRNA_intronic	1.46	5	**2**					CEBPB,CEBPD,Zbtb3	F	F	T
rs8142907	0.53	39390968	C	T	0.50	0.03	0.09	0.49	-0.09	0.07	ncRNA_intronic	1.97	7	**2**					6 altered motifs	F	F	T
rs2142833	0.53	39392296	A	G	0.50	0.03	0.09	0.49	-0.09	0.07	ncRNA_intronic	4.12	**1c**	**7**		ESC, IPSC, BLD	BLD	PU1	12 altered motifs	T	T	T
rs6001363	0.53	39392480	T	C	0.50	0.03	0.09	0.49	-0.09	0.07	ncRNA_intronic	2.21	**1f**	**4**		4 tissues			Maf,Nr2f2,Nrf-2	T	F	T
rs6001364	0.84	39393332	A	C	0.34	-0.06	6.02×10^{-4}	0.32	-0.13	0.01	ncRNA_intronic	2.20	**1f**	**7**	SKIN	IPSC, ESC, BLD	MUS		ZBTB7A, ZNF219	T	F	T

© 2024 XU HM et al. *JAMA Network Open.*

Candidate variant	r^2	Pos	EA	RA	GWAS results[b]						Annovar Function	CADD PHRED[c]	RDB Score[d]	Prioritization score[e]	HaploReg Evidence					RDB evidence		
					SIT			CKB														
					MAF	Beta	*P*-value	MAF	Beta	*P*-value					Promoter Histone Marks	Enhancer histone marks	DNase	Proteins bound	Motifs changed	eQTLs	TF binding	DNase peak
rs35043274	0.54	39394655	A	G	N.A.	N.A.	N.A.	0.24	-0.16	0.008	upstream	5.00	7	**2**					STAT	F	F	F
rs12159666	0.84	39395144	G	A	0.34	-0.06	7.95×10^{-4}	0.32	-0.13	0.01	upstream	0.13	6	**3**					4 altered motifs	F	F	F
rs9607600	0.53	39395418	T	C	0.50	0.03	0.11	0.49	-0.09	0.08	intergenic	0.004	5	**5**		ESC, IPSC	ESDR,ESC		Hsf	F	F	T
rs9611069	0.84	39395612	A	G	0.34	-0.06	6.59×10^{-4}	0.32	-0.13	0.01	intergenic	2.18	6	**6**		ESC, IPSC	MUS,LIV		4 altered motifs	T	F	T
rs9611070	0.53	39395707	T	G	0.50	0.03	0.11	0.49	-0.09	0.08	intergenic	2.15	6	**4**		ESC, IPSC			4 altered motifs	T	F	T
rs9611072	0.53	39397193	G	C	0.50	0.03	0.12	0.49	-0.09	0.08	intergenic	2.71	7	**2**					CTCF	F	F	T
rs28407607	0.55	39399079	A	G	N.A.	N.A.	N.A.	0.24	-0.16	0.009	intergenic	0.30	**3a**	**6**		11 tissues	ESC,BLD,BLD		4 altered motifs	F	T	T
**rs9607601**	**0.87**	**39399438**	**T**	**C**	**0.34**	**-0.06**	**5.11×10^{-4}**	**0.32**	**-0.14**	**0.009**	**intergenic**	**7.71**	**1b**	**10**	**SKIN, LIV**	**19 tissues**	**46 tissues**	**9 bound proteins**	**6 altered motifs**	**T**	**T**	**T**
rs4315626	0.55	39399738	C	T	0.50	0.03	0.13	0.49	-0.11	0.04	intergenic	0.47	5	**5**		12 tissues	BLD,BLD,BLD		8 altered motifs	F	T	T
rs9611074	0.56	39400343	G	A	N.A.	N.A.	N.A.	0.25	-0.16	0.009	intergenic	5.20	6	**3**					10 altered motifs	F	F	T
rs6001367	0.87	39400527	A	G	0.34	-0.06	5.18×10^{-4}	0.32	-0.14	0.01	intergenic	0.87	7	**4**					Mef2	F	F	T
rs6001368	0.87	39400832	A	G	0.34	-0.06	4.98×10^{-4}	0.32	-0.14	0.01	intergenic	0.89	7	**4**					4 altered motifs	F	F	T
rs6519161	0.88	39402450	T	C	0.34	-0.06	8.27×10^{-4}	0.32	-0.13	0.01	intergenic	1.00	7	**3**					ERalpha-a	F	F	F
rs9611075	0.57	39402505	A	G	N.A.	N.A.	N.A.	0.24	-0.16	0.01	intergenic	0.33	7	**2**					Hic1,TCF12,ZEB1	F	F	F
rs5995652	0.55	39404155	A	G	0.50	0.03	0.14	0.49	-0.10	0.049	intergenic	0.12	5	**4**		BLD			5 altered motifs	F	T	T
rs5995653	0.88	39404249	A	G	0.34	-0.06	5.16×10^{-4}	0.32	-0.14	0.01	intergenic	1.79	5	**6**		BLD	BLD		Foxp1	F	T	T
rs6001370	0.88	39404635	G	A	0.34	-0.06	6.96×10^{-4}	0.32	-0.13	0.02	intergenic	7.99	5	**7**		BLD	BLD		DMRT2	F	T	T
rs9611077	0.79	39405179	C	A	N.A.	N.A.	N.A.	0.29	-0.13	0.03	intergenic	1.86	6	**3**					16 altered motifs	F	F	T
**rs5995654**	**1.00**	**39405511**	**A**	**G**	**0.35**	**-0.06**	**1.17×10^{-5}**	**0.32**	**-0.14**	**0.009**	**intergenic**	**0.36**	**1f**	**3**					**HDAC2**	**T**	**F**	**T**

© 2024 XU HM et al. *JAMA Network Open.*

Candidate variant	r^2	Pos	EA	RA	GWAS results[b]						Annovar Function	CADD PHRED[c]	RDB Score[d]	Prioritization score[e]	HaploReg Evidence					RDB evidence		
					SIT			CKB														
					MAF	Beta	*P*-value	MAF	Beta	*P*-value					Promoter Histone Marks	Enhancer histone marks	DNase	Proteins bound	Motifs changed	eQTLs	TF binding	DNase peak
rs6001371	0.56	39405707	G	A	N.A.	N.A.	N.A.	0.47	-0.10	0.06	intergenic	2.18	6	**3**					5 altered motifs	T	F	T
rs6001372	0.76	39405942	T	C	0.39	-0.03	0.04	0.37	-0.11	0.03	intergenic	0.48	6	**2**					Zfx	F	F	T
rs6001373	0.75	39406143	T	A	0.39	-0.04	0.04	0.37	-0.11	0.03	intergenic	0.37	6	**3**					12 altered motifs	T	F	T
rs7289061	0.93	39406322	A	G	0.32	-0.05	4.22×10^{-5}	0.30	-0.16	0.004	intergenic	0.30	6	**2**					7 altered motifs	F	F	T
rs7289064	0.55	39406330	A	G	N.A.	N.A.	N.A.	0.47	-0.09	0.08	intergenic	0.31	7	**1**					4 altered motifs	F	F	F
rs2413562	0.76	39406711	A	G	0.39	-0.03	0.05	0.37	-0.11	0.03	intergenic	0.54	7	**4**		LIV			ATF3	F	T	T
rs6001374	0.76	39406881	T	A	0.39	-0.03	0.06	0.37	-0.11	0.03	intergenic	1.49	**1f**	**6**		ESC, LIV, BLD	IPSC		ATF3	T	T	T
rs6001375	0.93	39407116	A	G	0.32	-0.06	5.85×10^{-4}	0.30	-0.16	0.004	intergenic	0.12	7	**4**		4 tissues			Rad21	F	T	T
rs6001376	0.75	39407399	C	T	0.39	-0.03	0.09	0.37	-0.11	0.04	intergenic	0.54	**1f**	**6**		4 tissues	7 tissues	CTCF, RAD21, EGR1		T	T	T
rs4256060	0.76	39407546	T	G	0.39	-0.03	0.06	0.37	-0.11	0.04	intergenic	1.56	**4**	**4**		5 tissues			8 altered motifs	F	T	T
rs4299420	0.93	39407685	G	T	0.32	-0.06	0.001	0.30	-0.15	0.007	intergenic	6.57	**4**	**4**		4 tissues			GR	F	T	F
rs6001379	0.91	39408307	G	A	N.A.	N.A.	N.A.	0.30	-0.15	0.009	intergenic	2.44	6	**5**		BLD, LIV			Ets,NERF1a	F	T	T
rs4443098	0.75	39408841	A	G	N.A.	N.A.	N.A.	0.36	-0.09	0.09	intergenic	0.33	**1b**	**8**	BLD	9 tissues	BLD	CTCF,SMC3	6 altered motifs	T	T	T

© 2024 XU HM et al. *JAMA Network Open.*

[a] 45 candidate SNPs in the genomic locus of rs5995654, including all SNPs that are within 250Kb distance and in linkage disequilibrium (r^2>0.4) with rs5995654. The independent SNP rs5995654 is bolded, and rs9607601 with the strongest priority evidence is marked red.
[b] GWAS results for gastric lesion progression in SIT cohort and for GC incident risk in CKB cohort.
[c] CADD score computed based on 63 annotations. A higher score indicates more deleterious effect.
[d] RegulomeDB (RDB) score ranging from 1a to 7. 1a stands for the highest score for SNPs with the most biological evidence to be a regulatory element.
[e] Variant prioritization was performed incorporating five functional annotation terms via HaploReg (v4.1, Haploreg Promoter Histone Marks, Haploreg Enhancer Histone marks, Haploreg DNase, Haploreg Proteins bound, Haploreg Motifs changed), three terms via Regulome DB (v2.1, RDB eQTLs, TF binding, DNase peak) and two terms on stomach-based and blood-based eQTLs. Prioritization score integrating these ten terms was calculated for the candidate variants (within±250Kb and r^2>0.4 of independent variant), with a score of 1 assigned for each term. RDB evidence of eQTLs, TF binding and DNase peak was displayed as "T" (true) or "F" (false), while significant stomach-based and blood-based eQTLs (*P*<0.05) were shown as "+", otherwise "-" in the table.
Abbreviations: Chr, chromosome; CKB, China Kadoorie Biobank; EA, effect allele; eQTL, expression quantitative trait loci; GWAS, genome-wide association study; MAF, minor allele frequency; Pos, Position; RA, reference allele; SIT, Shandong Intervention Trial.

© 2024 XU HM et al. *JAMA Network Open.*

eTable 14. Gene-Intervention interaction analysis for rs9607601.

rs9607601	CC genotype		CT/TT genotype		*P* for interaction
	No. of case / Subjects	HR (95% CI)[a]	No. of case / Subjects	HR (95% CI)[a]	
H. pylori treatment					
Placebo	36/390	Ref	30/511	Ref	**0.002**
Active	12/371	**0.35 (0.18-0.67)**	28/498	0.94 (0.56-1.56)	
Vitamin supplementation					
Placebo	35/550	Ref	38/717	Ref	0.53
Active	30/537	1.02 (0.58-1.82)	28/682	0.81 (0.48-1.36)	
Garlic supplementation					
Placebo	32/553	Ref	33/534	Ref	0.19
Active	41/615	1.17 (0.66-2.08)	25/684	0.72 (0.42-1.22)	

[a] Analyses were conducted using Fine-Gray regression models adjusting for age, sex, *H. pylori* infection status, and principal components.
Abbreviations: CI, confidence interval; *H.pylori*, *Helicobacter pylori*; HR, hazard ratio.

© 2024 XU HM et al. *JAMA Network Open.*

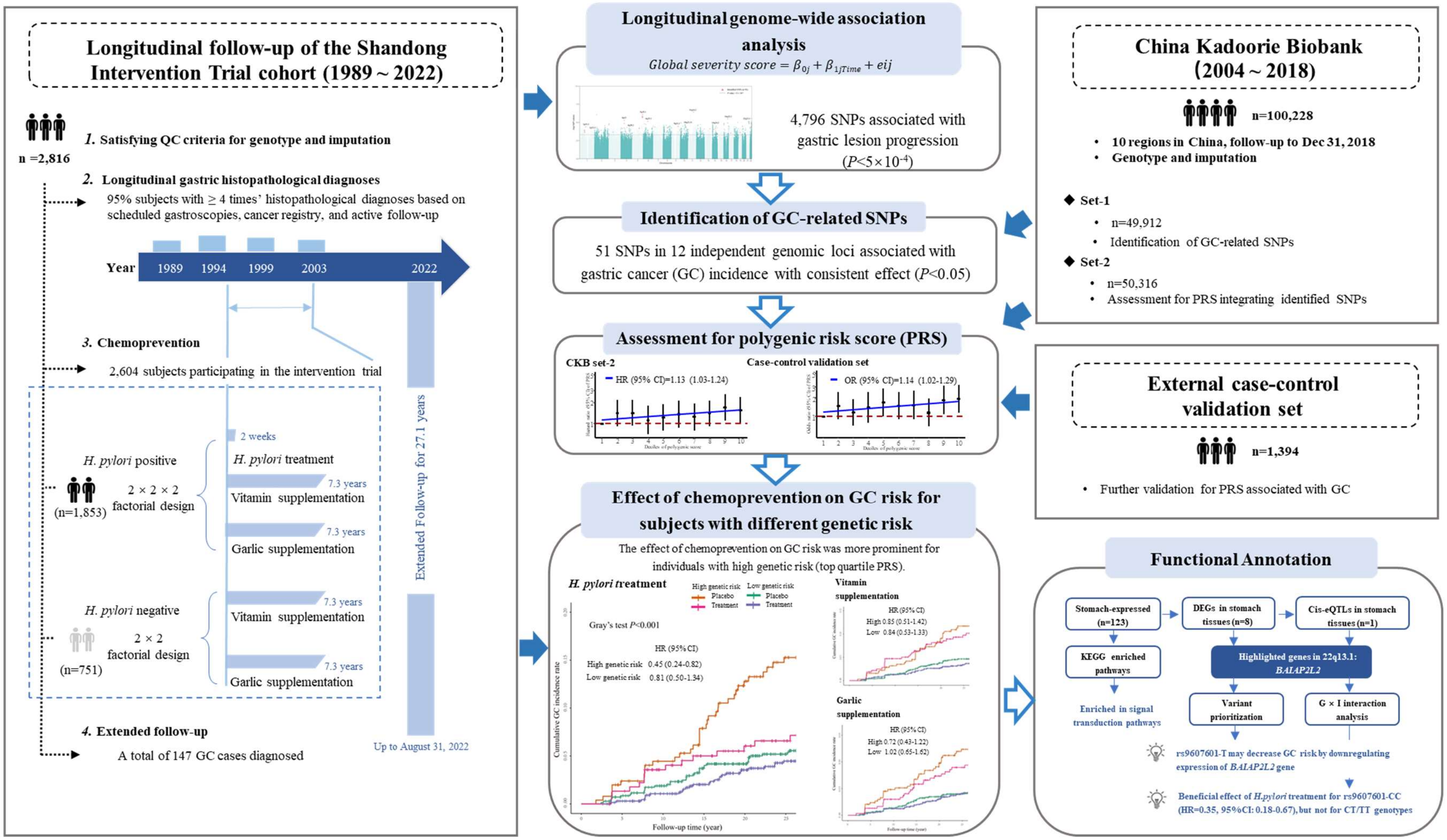

eFigure 1. Study design. Abbreviations: CI, confidence interval; DEGs, differentially expressed genes; eQTL, expression quantitative trait loci; GC, gastric cancer; *H.pylori*, *Helicobacter pylori*; HR, hazard ratio; OR, odds ratio; PRS, polygenic risk score; QC, quality control; SNP, single nucleotide polymorphism.

© 2024 XU HM et al. *JAMA Network Open.*

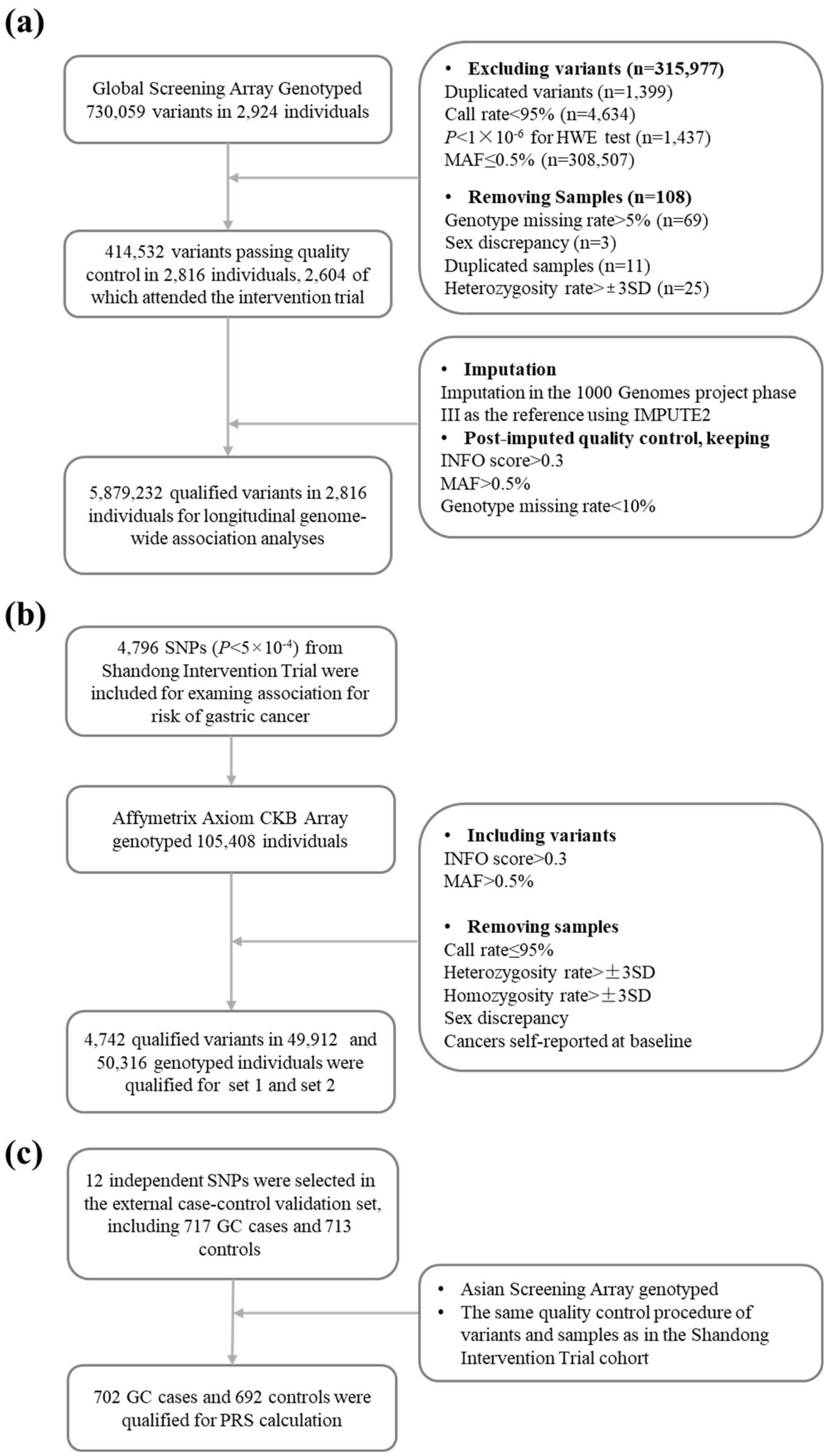

eFigure 2. Quality control for selection of genetic variants. (a) Quality Control in the Shandong Intervention Trial cohort (b) Quality Control in the China Kadooire Biobank cohort (c) Quality Control in the external case-control validation study. Abbreviations: HWE, Hardy–Weinberg Equilibrium; MAF, minor allele frequency; PRS, polygenic risk score; SD, standard deviation.

© 2024 XU HM et al. *JAMA Network Open.*

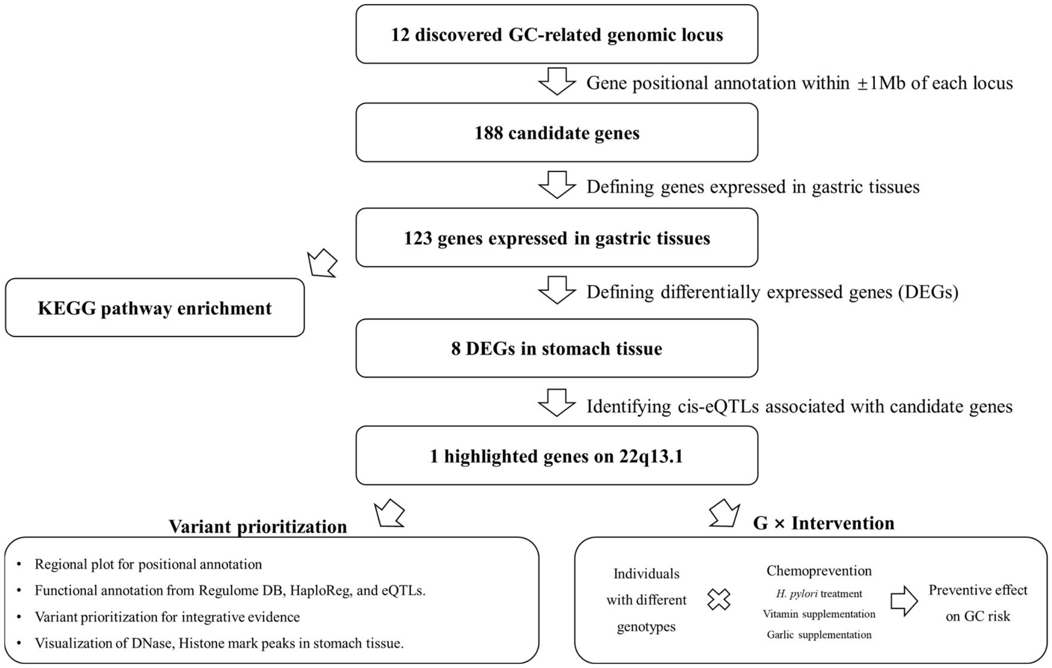

eFigure 3. Flow diagram of functional annotation. Abbreviations: DEGs, differentially expressed genes; eQTL, expression quantitative trait loci; GC, gastric cancer; *H. pylori*, *Helicobacter pylori;* LD, linkage disequilibrium.

© 2024 XU HM et al. *JAMA Network Open.*

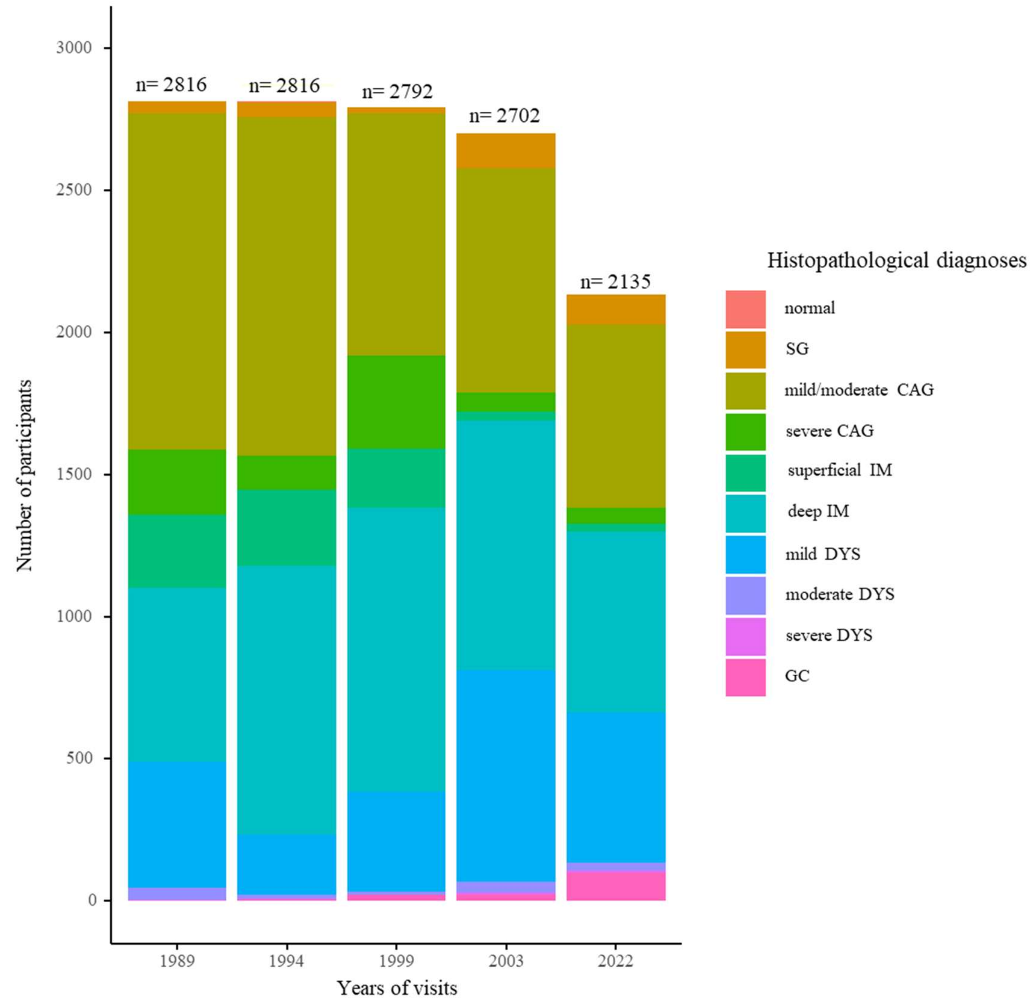

eFigure 4. Gastric histopathology of the Shandong Intervention Trial participants. Abbreviations: CAG, chronic atrophic gastritis; DYS, dysplasia; GC, gastric cancer; IM, intestinal metaplasia; SG, superficial gastritis.

© 2024 XU HM et al. *JAMA Network Open.*

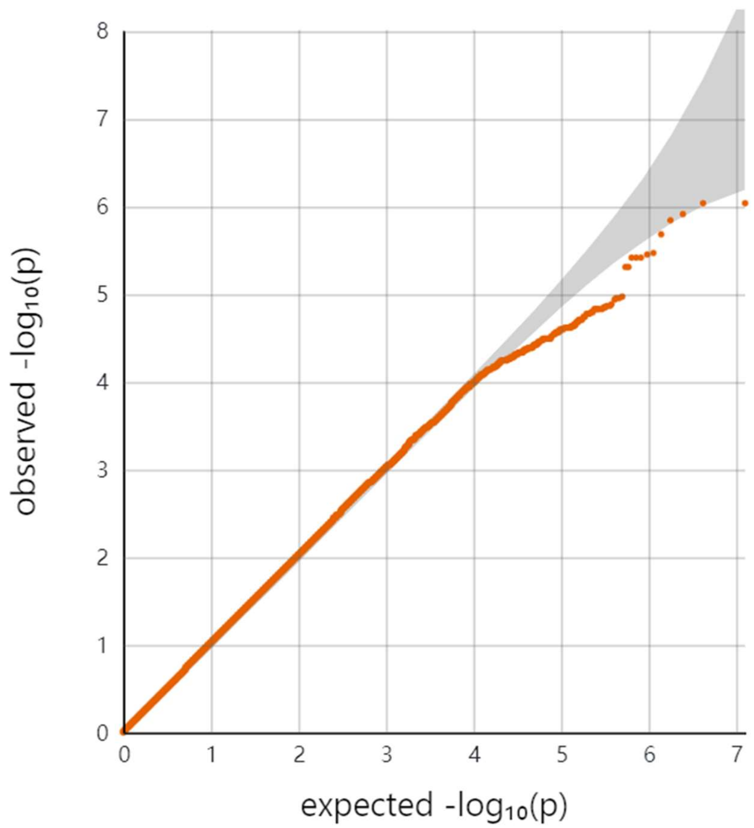

eFigure 5. Q-Q plot for longitudinal genome-wide association analysis. The orange circles represent the distribution of *P* values for associations in the Shandong Intervention Trial cohort. The observed versus expected χ2 test statistics shows no evidence for inflation of the χ2 test. Genomic inflation actor (λ): 1.037.

© 2024 XU HM et al. *JAMA Network Open.*

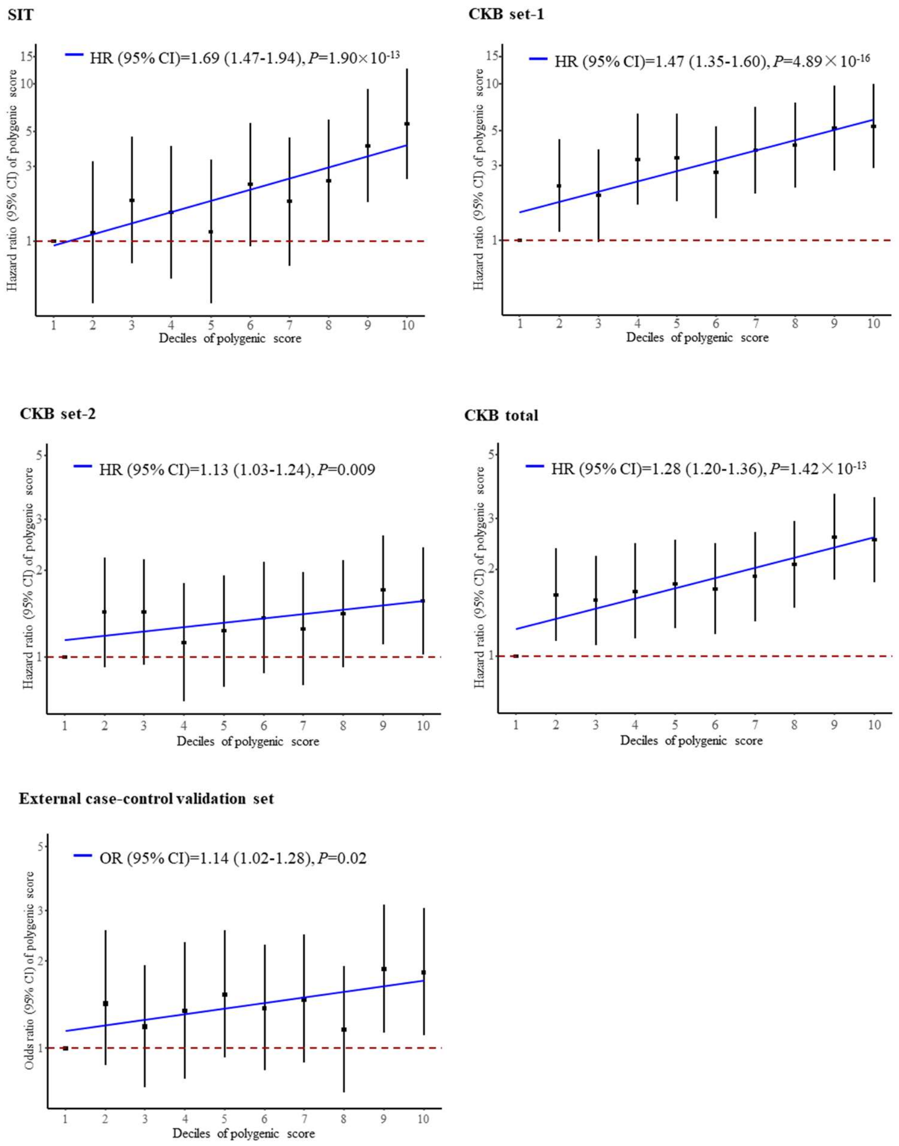

eFigure 6. The associations of polygenic risk scores with the risk of incident gastric cancer. Participants were divided into deciles based on their polygenic risk scores in the SIT cohort, CKB set-1, CKB set-2, total CKB cohort, and the external case-control validation set, respectively. For SIT and CKB cohort, the Fine-Gray proportional subdistribution hazards models were used for analysis accounting for death from causes other than gastric cancer as competing risk, adjusting for age, sex, *H. pylori* infection (for SIT only), regions (for CKB only), and principal components. For the case-control validation set, the logistic regression model was used adjusting for age, sex, *H. pylori* infection and principal components. The HRs (ORs) for each genetic group were calculated with decile 1 of the polygenic risk score as reference (HR or OR=1.00), and the 95% CI is shown by the error bars. The blue lines depict the association between polygenic risk score (per standard deviation) and the risk of gastric cancer. Abbreviations: CI, confidence interval; CKB, China Kadoorie Biobank; HR, hazard ratio; OR, odds ratio; SIT, Shandong Intervention Trial.

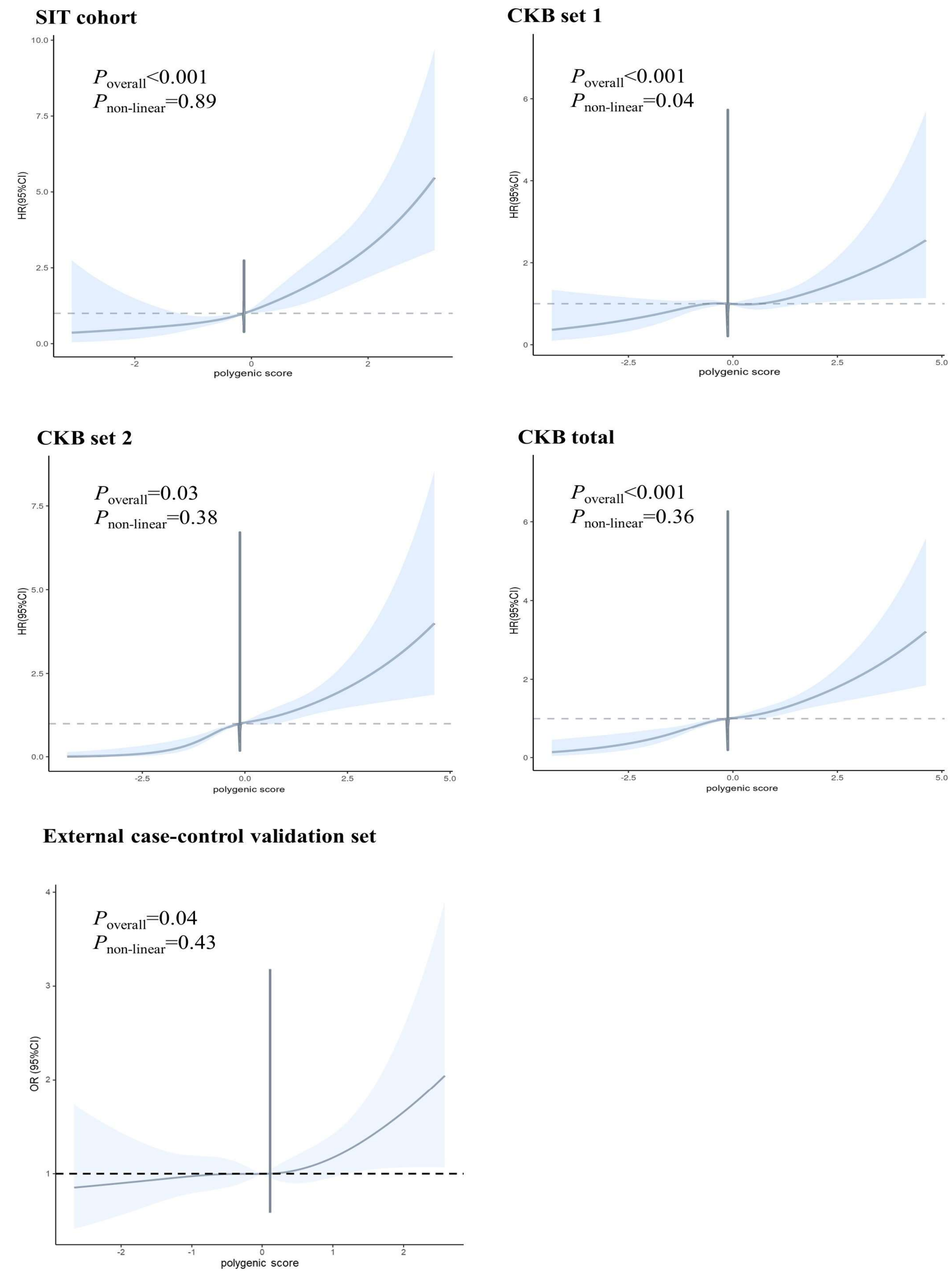

eFigure 7. Restricted cubic spline curves of polygenic risk score and incident gastric cancer risk. Restricted cubic spline models were used for examining the nonlinear relationship between polygenic risk score and gastric cancer. For SIT and CKB cohort, Fine-Gray model was applied adjusting for age, sex, *H. pylori* infection status (for SIT only), regions (for CKB only), and principal components. For the external case-control validation set, logistic regression model was used, adjusting for age, sex, *H. pylori* infection status and principal components. Abbreviations: CKB, China Kadoorie Biobank; SIT, Shandong Intervention Trial.

© 2024 XU HM et al. *JAMA Network Open.*

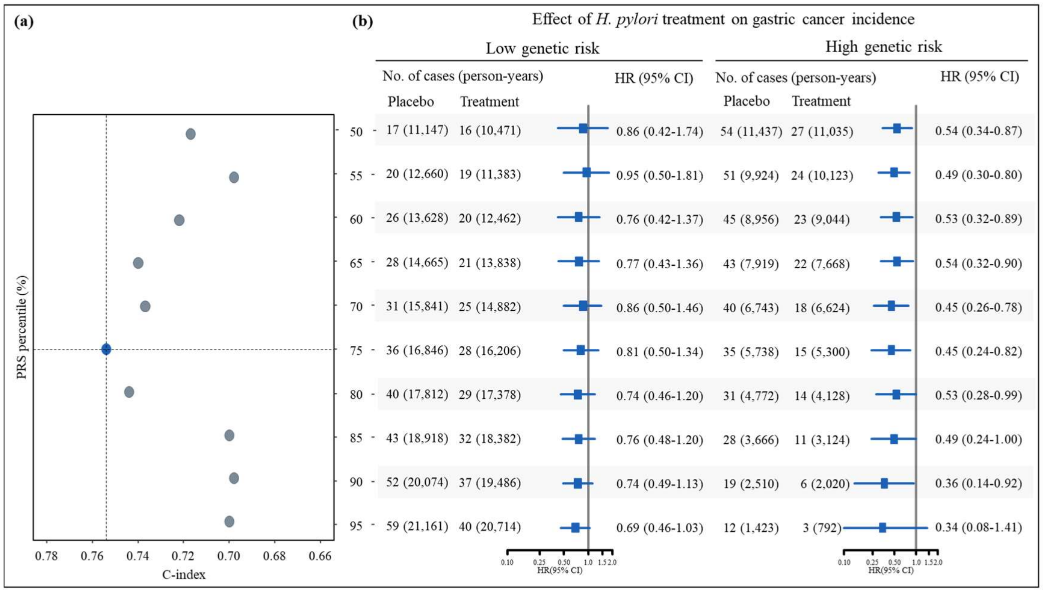

eFigure 8. Determination of PRS cut-off percentile for genetic-risk classification and the association of *H.pylori* treatment with GC incidence. (a) Determination of the PRS cut-off percentile for genetic-risk classification based on the gradient boosting method. We calculated C-index under PRS cut-off thresholds incremented by 5%, spanning from 50% to 95%. The 75th percentile of PRS with the highest C-index (0.754) is highlighted with blue color. (b) The effect of *H. pylori* treatment on gastric cancer in the respective low and high genetic risk group. Individuals having the PRS above each percentile threshold were defined as having a high genetic risk and otherwise having a low genetic risk. The HRs (95% CIs) were computed using the Fine-Gray proportional subdistribution hazards models, which account for death from causes other than gastric cancer as a competing risk, adjusting for age, sex, and principal components.

Abbreviations: CI, confidence interval; GC, gastric cancer, HR, hazard ratio; PRS, polygenic risk score.

© 2024 XU HM et al. *JAMA Network Open.*

	No. of cases (person-years)			HR (95% CI)	ARR (%)	NNT	*P* for interaction
	Placebo	Treatment					
***H. pylori* positive**							
Vitamin supplementation							
High genetic risk	24 (5,658)	26 (5,380)		1.09 (0.62-1.91)	7.75	12.90	0.51
Low genetic risk	36 (16,630)	28 (16,422)		0.84 (0.51-1.38)	1.05	95.24	
Garlic supplementation							
High genetic risk	28 (5,483)	22 (5,555)		0.81 (0.45-1.47)	1.41	71.05	0.43
Low genetic risk	32 (16,724)	32 (16,328)		1.05 (0.64-1.72)	1.20	83.28	
***H. pylori* negative**							
Vitamin supplementation							
High genetic risk	10 (1,802)	4 (2,013)		0.37 (0.10-1.32)	8.54	11.71	0.29
Low genetic risk	6 (7,233)	5 (6,823)		1.03 (0.31-3.39)	0.26	378.23	
Garlic supplementation							
High genetic risk	10 (2,263)	4 (1,552)		0.51 (0.15-1.71)	4.36	22.95	0.54
Low genetic risk	6 (6,767)	5 (7,289)		0.90 (0.26-3.10)	0.53	188.23	

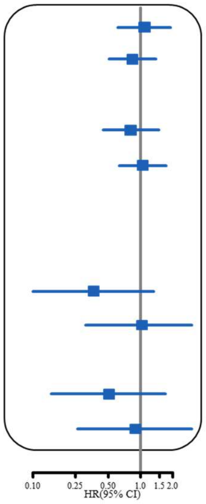

eFigure 9. The association of nutrition supplementation with gastric cancer for low or high genetic-risk stratified by *H. pylori* infection. The HRs (95% CIs) were computed using the Fine-Gray proportional subdistribution hazards models, which account for death from causes other than gastric cancer as a competing risk, while adjusting for age, sex, and principal components. ARR and NNT were calculated for the entire follow-up period (27.1 years) of the SIT. *P* values for interaction were obtained by incorporating a multiplicative term of the examined intervention and genetic risk variables into the Fine-Gray models. Abbreviations: ARR, absolute risk reduction; CI, confidence interval; *H. pylori*, *Helicobacter pylori*; HR, hazard ratio; NNT, number needed to treat; SIT, Shandong Intervention Trial.

© 2024 XU HM et al. *JAMA Network Open.*

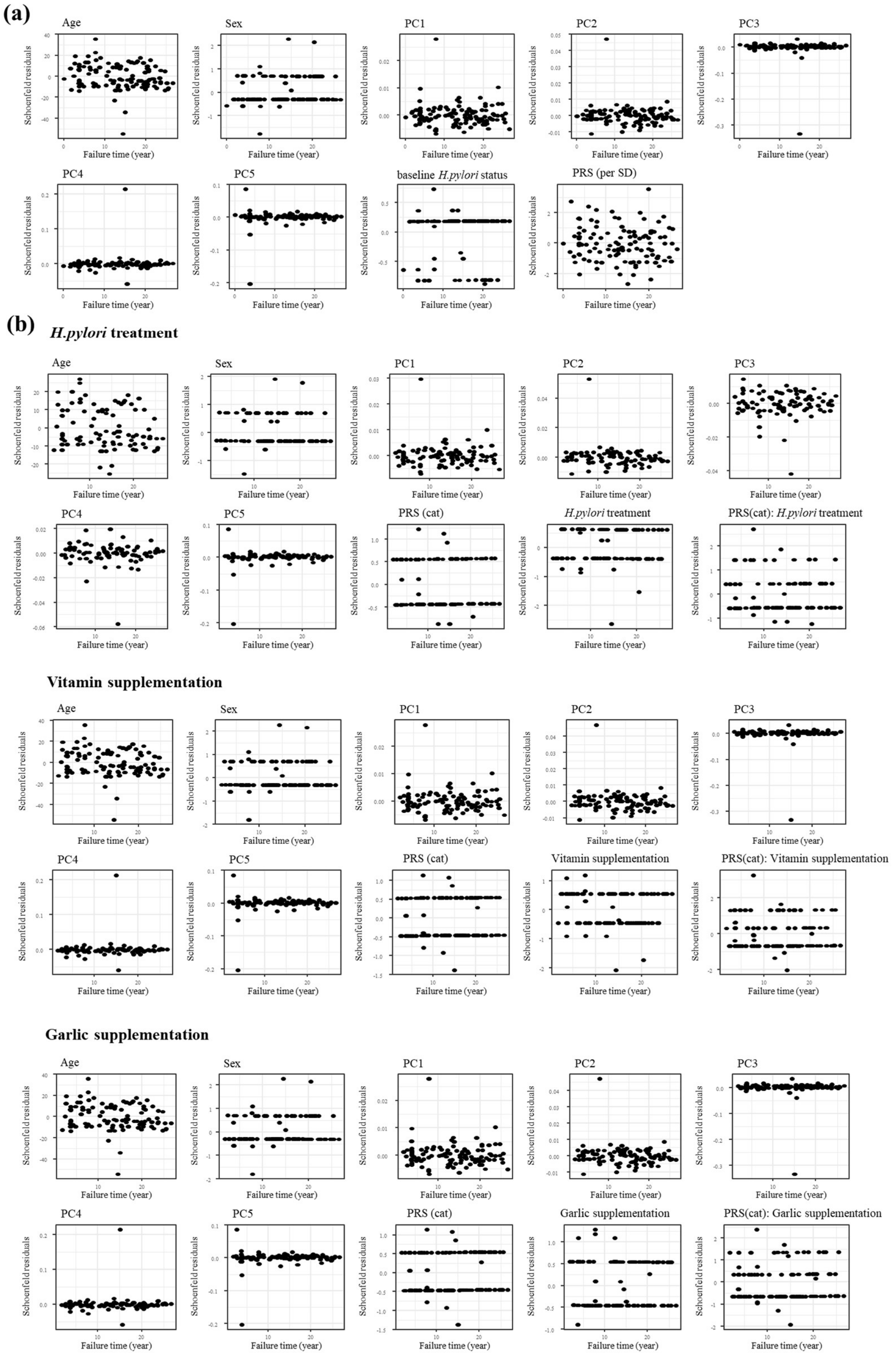

eFigure 10. Schoenfeld residuals test for using Fine-Gray models. Proportional subdistribution hazard assumption was tested utilizing Schoenfeld residuals methods in analyses of (a) association between polygenic risk score and gastric cancer incidence; and (b) the association of *H.pylori* treatment, vitamin supplementation, and garlic supplementation with gastric cancer risk by genetic-risk, and the interactions between genetic predisposition and interventions, adjusting for other covariates.

© 2024 XU HM et al. *JAMA Network Open.*

	Low genetic risk: No. of cases (person-years)	Low genetic risk: HR (95% CI)	Low genetic risk: ARR (%)	Low genetic risk: NNT	High genetic risk: No. of cases (person-years)	High genetic risk: HR (95% CI)	High genetic risk: ARR (%)	High genetic risk: NNT	*P* for interaction
No *H. pylori* treatment or vitamin supplementation	18 (8,718)	Ref			19 (2,899)	Ref			
H. pylori treatment only	18 (7,912)	1.09 (0.56-2.10)	0.55	183.19	5 (2,759)	0.28 (0.10-0.74)	11.56	8.65	0.03
Vitamin supplementation only	18 (8,128)	1.12 (0.58-2.17)	0.39	254.56	16 (2,838)	0.87 (0.43-1.77)	2.11	47.32	0.60
H. pylori treatment and vitamin supplementation	10 (8,294)	0.63 (0.29-1.36)	2.25	44.39	10 (2,542)	0.55 (0.25-1.21)	6.15	16.25	0.97

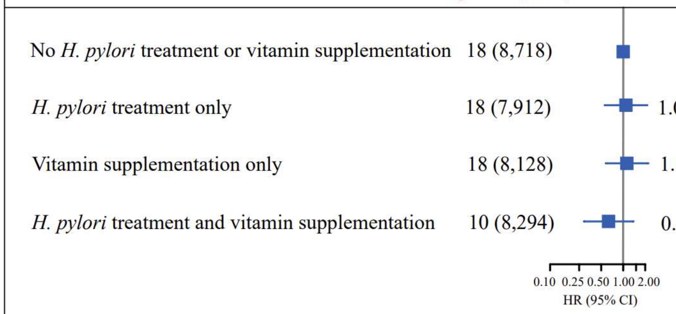

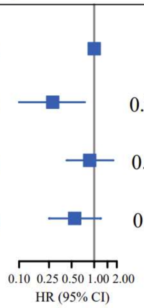

eFigure 11. The joint effect of *H. pylori* treatment and vitamin supplementation by genetic risk among *H. pylori*-positive subjects. The HRs (95% CIs) were calculated using the Fine-Gray proportional subdistribution hazards models accounting for death from causes other than gastric cancer as competing risk, adjusting for age, sex, *H.pylori* infection status and principal components, based on the Shandong Intervention Trial. ARR and NNT were measured for the entire follow-up period (27.1 years) of the Shandong Intervention Trial. *P* values for interaction were obtained by incorporating a multiplicative term of the examined intervention and genetic risk variables into the Fine-Gray models. Abbreviations: ARR, absolute risk reduction; CI, confidence interval; *H. pylori, Helicobacter pylori*; HR, hazard ratio; NNT, number needed to treat.

© 2024 XU HM et al. *JAMA Network Open.*

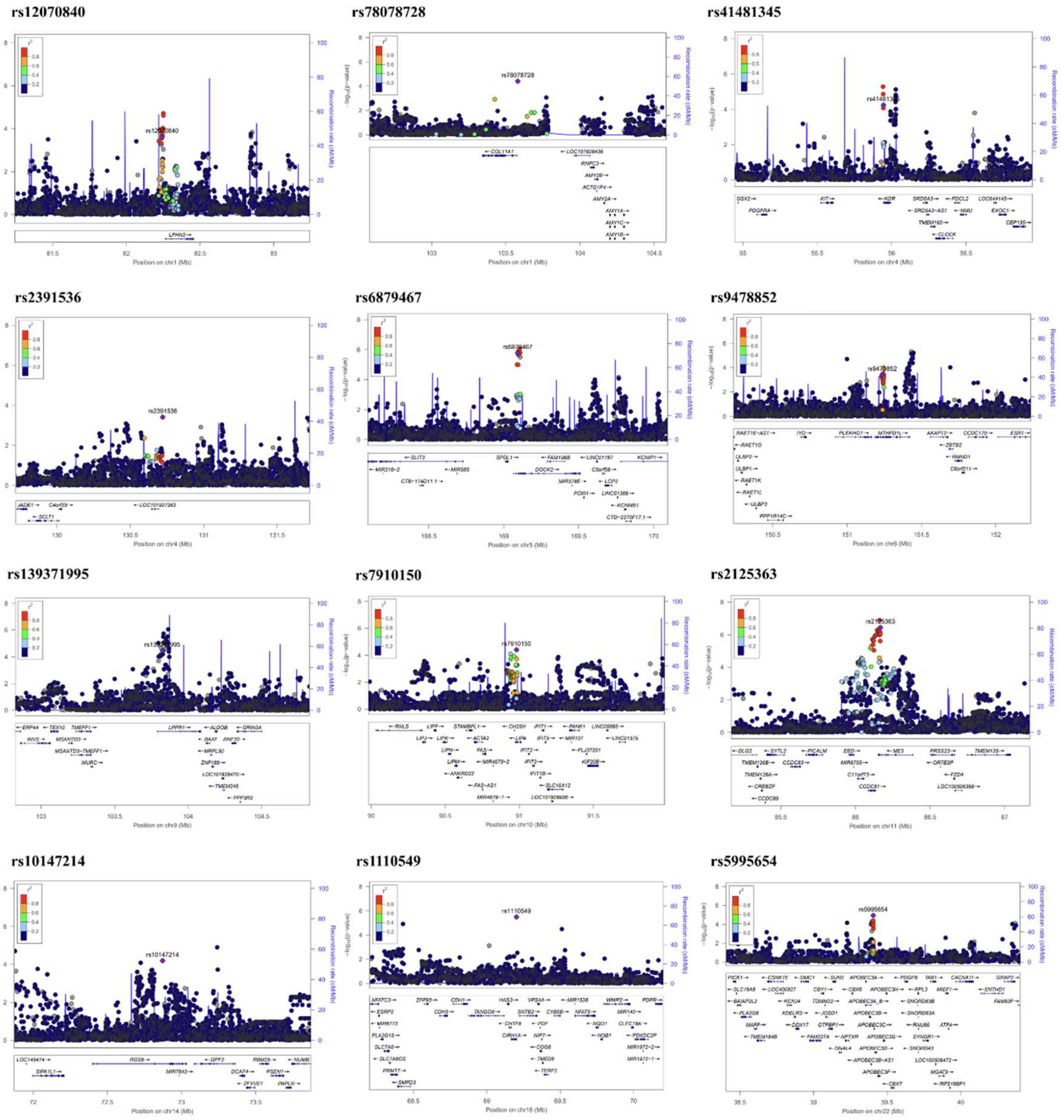

eFigure 12. Regional plots for the 12 genomic loci. Plots were produced in LocusZoom and show the most strongly associated SNP (purple diamond) with *P* values derived from the genome-wide association study, with the color of each SNP representing the linkage disequilibrium to the lead SNPs.

© 2024 XU HM et al. *JAMA Network Open.*

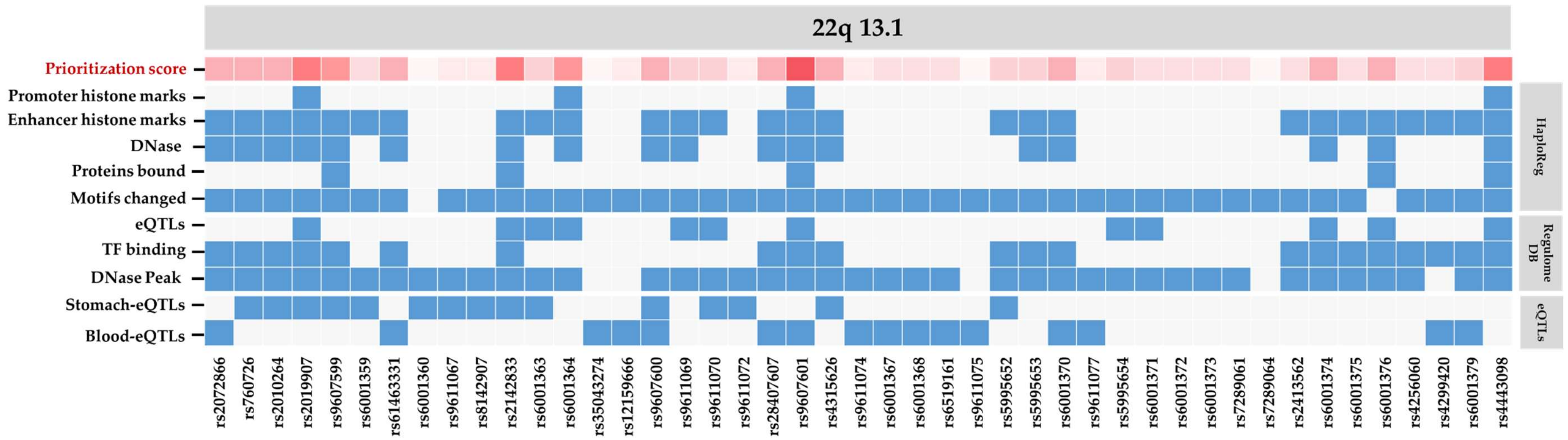

eFigure 13. Variant prioritization. Variant prioritization was performed incorporating five functional annotation terms via HaploReg (v4.1, Haploreg Promoter Histone Marks, Haploreg Enhancer histone marks, Haploreg DNase, Haploreg Proteins bound, Haploreg Motifs changed), three terms via Regulome DB (v2.1, RegulomeDB eQTLs, TF binding, DNase peak) and two terms on stomach-based and blood-based eQTLs. Prioritization score integrating these ten terms was calculated for the candidate variants (within ±250Kb and r^2>0.4 with the lead variant [rs5995654] of this genomic region), with a score of 1 assigned for each term. In this plot, each blue chunk represents an annotated indicator of each SNP, and the gradient red chunk above represents the prioritization score (0-10). Abbreviations: eQTL, expression quantitative trait loci; SNP, single nucleotide polymorphism; TF, transcription factor.

© 2024 XU HM et al. *JAMA Network Open.*

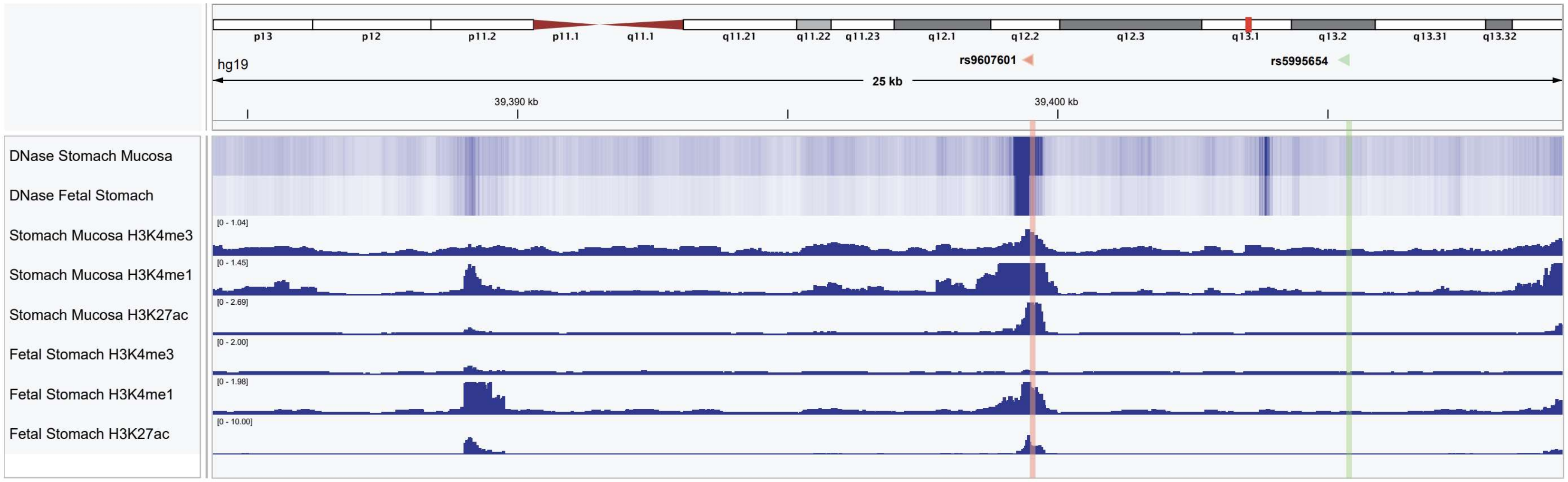

eFigure 14. Roadmap annotation of 22q13.1. ChIP-Seq data show enrichments of histone marks and DNase I hypersensitivity sites in the region of rs9607601 in strong LD with the lead variant rs5995654 (r^2 =0.87). Abbreviations: ChIP, chromatin immunoprecipitation; LD linkage disequilibrium.

© 2024 XU HM et al. *JAMA Network Open.*
