# Supplementary material for: Helicobacter pylori Treatment and Gastric Cancer Risk Among Individuals With High Genetic Risk for Gastric Cancer
Source: JAMA Netw Open. 2024 May 29;7(5):e2413708. doi: 10.1001/jamanetworkopen.2024.13708 (PMC11137637; doi:10.1001/jamanetworkopen.2024.13708)
Supplement: Supplement 2. — Data Sharing Statement [file jamanetwopen-e2413708-s002.pdf]

Data Sharing Statement

Xu. Helicobacter pylori Treatment and Gastric Cancer Risk Among Individuals With High Genetic Risk for Gastric Cancer. *JAMA Netw Open*. Published May 29, 2024. doi:10.1001/jamanetworkopen.2024.13708

Data
Data available: No

Additional Information
Explanation for why data not available: The data of the Shandong Intervention Trial, including the original trial and continuous follow-up can be made available upon acceptance of an official request to the corresponding author. Data Sharing details of how to access China Kadoorie Biobank data and details of the data release schedule are available from www.ckbiobank.org/site/Data+Access.
